# Integrated multi-tissue transcriptomics reveals cross-tissue regulatory networks and hub genes regulating feed efficiency in aging chicken

**DOI:** 10.1016/j.psj.2025.105711

**Published:** 2025-08-21

**Authors:** Yuejie Han, Fangren Lan, Ronglang Cai, Wenxin Zhang, Daqing Dai, Xinwei Jiang, Junnan Zhang, Ning Yang, Congjiao Sun

**Affiliations:** a State Key Laboratory of Animal Biotech Breeding, Frontier Science Center of Molecular Design Breeding, China Agricultural University, Beijing 100193, China; b National Engineering Laboratory for Animal Breeding, College of Animal Science and Technology, China Agricultural University, Beijing 100193, China

**Keywords:** Feed efficiency, Multi-tissue transcriptomics, Lipid metabolism, Hub genes, Chicken

## Abstract

Given the critical impact of feed costs on poultry profitability, it is essential to improve feed efficiency through genetic selection. Although residual feed intake (**RFI**) serves as the gold-standard metric for feed efficiency assessment, its molecular regulation remains poorly understood. This study estimated the residual feed intake of laying hens from 70 to 100 weeks of age (**E-RFI**) and estimated the genetic parameters of key production traits (egg quality and feed efficiency) and lipid traits (triglycerides: TG; free fatty acids: FFA; cholesterol: TC). By decoupling the genetic covariance with production traits, we selected 40 extreme E-RFI individuals and performed multiple aspects of transcriptomic analysis across seven tissues (hypothalamus, pituitary, liver, pancreas, duodenum, ileum, and cecum). Key findings revealed moderate heritability of E-RFI (0.45), with production traits showing distinct genetic potential. Egg number (**EN_70-100w_**, 0.37) and average egg weight (**AEW_70-100w_**, 0.64) for 70 to 100 weeks of age exhibited moderate and high heritability, respectively. Lipid traits displayed low to moderate heritability (TG: 0.01-0.19; FFA: 0.15-0.17; TC: 0.08). Enhanced feed efficiency (reduced E-RFI) correlated with increased egg weight and yolk lipid content but decreased yolk mass and dry matter (DM). Concurrently, hepatic DM (+), FFA (+), and TC (-) levels shifted, while abdominal fat deposition (weight, dry matter, lipids) increased, mirroring yolk lipid dynamics. A total of 22 hub genes (including *SHMT1, OAT, CPT1A,* and *CYCS*) were identified associated with E-RFI through multi-tissue integrative analysis, which were specifically enriched in the liver, duodenum, and cecum tissues and exhibited significant cross-tissue functional synergy. These genes constructed a multidimensional regulatory network that influenced feed efficiency through specific molecular interaction mechanisms. Functional analysis revealed that these genes are primarily involved in key biological processes, such as energy metabolism, protein homeostasis regulation, immune signal transduction, and amino acid/lipid metabolism, and regulate growth performance and feed utilization of laying hens through multiple pathways. These findings established a molecular framework for optimizing feed efficiency in aging layers using targeted genetic strategies.

## Introduction

The sustainable development of livestock production has become a central industry priority, driven by the growing demand for animal products, resource optimization imperatives, and environmental protection requirements. Consequently, modern poultry breeding primarily aims to achieve cost-effective production of meat and eggs while maintaining operational efficiency(Van Eerden et al., 2004). To reconcile economic viability with production outputs, extending the laying cycle of egg-type chickens has emerged as an essential strategy for egg production(Bain et al., 2016). With the aging of hens, their physiological functions gradually decline, resulting in reduced feed efficiency, weakened production performance, and abnormal lipid metabolism(Liu et al., 2023; Song et al., 2021). Given that feed expenses constitute 60-70 % of total poultry production costs, improving feed efficiency during the late laying period has therefore become a primary focus in breeding(Sharma et al., 2018; Yang, 2020).

Residual feed intake (**RFI**), first established by Koch et al. as the difference between observed feed consumption and predicted requirements for growth and maintenance in a specific period(Koch et al., 1963), has been widely recognized as the most sensitive and accurate parameter for assessing feed efficiency. This heritable trait (*h²* = 0.3-0.5) demonstrates significant potential for genetic enhancement, especially through genomic selection strategies targeting associated genes or genetic variants(Yi et al., 2015). Karimi et al. identified RFI-associated lncRNAs and their target genes through integrated RNA-seq and bioinformatics analysis(Karimi et al., 2022). A transcriptome analysis of chicken duodenum revealed key RFI candidate genes (*MYO1D, MYO1E, MYO1A, USH1C,* and *EZR*), primarily functioning in intestinal villi structure and nutrient absorption(Xiao et al., 2021). Furthermore, the developmental characteristics of the gizzard, liver, and cecum were found to significantly influence broiler feed efficiency. Collectively, these findings provide valuable insights into the biological basis of RFI variation(Huang et al., 2022).

However, as a complex quantitative trait, RFI’s polygenic nature involves coordinated regulation across multiple physiological systems(Chen et al., 2011; Yang et al., 2020). RFI can be affected by physiological processes, such as appetite regulation, cellular activity and fat metabolism(Montanholi et al., 2013; Reyer et al., 2017; Xu et al., 2016). In addition, RFI exhibits inherent methodological limitations. As a trait calculated through multivariate regression models accounting for metabolic body weight and production output, RFI inherently incorporates genetic variance components associated with production traits(Kennedy et al., 1993). Consequently, identical RFI values in laying hens may mask the underlying genetic heterogeneity, particularly when production parameters (e.g., egg yield) differ substantially between individuals. Traditional genetic analyses of extreme-RFI populations predominantly focus on the trait itself, frequently overlooking the covariance between RFI-associated loci and production-related quantitative trait loci (**QTLs**). Emerging evidence reveals significant genetic correlations between RFI and key production characteristics, such as growth rate, demonstrating that RFI-associated genomic regions are pleiotropically linked to multiple traits (Saintilan et al., 2013). This interdependence necessitates integrated genomic approaches that simultaneously model RFI and production trait architecture. Therefore, the study of RFI and related production and lipid traits in the late stage of egg laying is particularly important. However, there are currently few studies on the late stage of egg laying, which cannot provide a sufficient and reliable theoretical basis for the selection of target traits in breeding work(Zhou et al., 2023).

This investigation focused on the RFI molecular mechanisms in 70-100-week-old laying hens, a critical yet understudied late-phase production window. Our novel experimental design implemented stratified randomization based on egg production parameters (*P* < 0.05), effectively decoupling genetic covariance between feed efficiency and reproductive traits. Multi-tissue transcriptome profiling spanning the neuroendocrine (hypothalamus, pituitary, etc.) and digestive systems was performed in age-matched high/low E-RFI cohorts (*n* = 10 per group). Systems-level functional enrichment analysis revealed inter-organ coordination patterns through Gene Ontology (**GO**) and Kyoto Encyclopedia of Genes and Genomes (**KEGG**) pathway mapping. Finally, significantly correlated functional gene modules were screened through weighted gene co-expression network analysis (**WGCNA**), and the cross-tissue co-regulatory mechanisms were elucidated along with the identification of relevant hub genes by integrating protein-protein interaction network analysis. This integrative approach delineates tissue-specific regulatory modules, while elucidating the systemic biological processes governing E-RFI variation. As the first multi-tissue gene regulatory atlas for late-phase laying hens, this study bridges critical knowledge gaps in persistent feed efficiency genetics and establishes a framework for precision breeding strategies that concurrently optimize productivity and metabolic efficiency.

## Materials and methods

### Ethics statement

The experimental procedures used in this study met the guidelines established by the Ministry of Agriculture of China for the care and use of experimental animals. The project was approved by the Institutional Animal Care and Use Committee of China Agricultural University, China (Issue No. 32303202-1-1).

### Animals, phenotypic data, and tissue sample collection

The entire procedure was performed according to the regulations and guidelines formulated by the Animal Care and Use Committee of China Agricultural University.

A total of 248 Rhode Island Red chickens from Beijing Huadu Yukou Poultry Breeding Co., Ltd. (China) were used in the current study. All chickens were from one batch and were reared in individual cages with free access to feed and water. The illumination schedule followed a photoperiod of 16 h light and 8 h darkness on a daily basis (16 L:8 D). Each hen was provided with mash feed on individual metal feeders.

The total egg production for each individual from the onset of laying to 100 weeks of age was counted. Egg weight (**EW**) and yolk weight (**YW**) were measured at 72, 80, 90, and 100 weeks of age, and the average values from these measurements were calculated to represent the average egg weight (**AEW_70-100w_**) and average yolk weight (**AYW_70-100w_**) at 70 to 100 weeks. The body weight (**BW**) of each chicken was measured at the 70th and 100th weeks of age using an electronic scale. Feed intake, egg mass, and body weight gain were recorded during two specific periods: from 70 to 71 weeks of age and from 99 to 100 weeks of age. Subsequently, the corresponding daily average feed intake (**DFI**), daily average egg production (**DEM**), and daily average body weight gain (**DBWG**) were determined. Finally, we calculated the total number of eggs for each chicken at the first 100 weeks of age (**TEN100**), the number of eggs for 70 to 100 weeks of age (**EN_70-100w_**), the average daily feed intake (**DFI_70-100w_**, estimated from the average daily feed intake of 70 to 71 weeks of age and 99 to 100 weeks of age), the average daily egg production (**DEM_70-100w_**, calculated from the average egg weight × egg number / days in this stage), and the average daily body weight gain (**DBWG_70-100w_**). Among them, the experimental group focused on the specific measurement stage of 70 to 100 weeks of age (about 490 to 700 days), and used 30 weeks (210 days) as the main time period of focus in this study.

The eggs laid by these 248 laying hens at their 100th week of age were collected and weighed. Egg yolk samples were collected and weighed using an egg white and egg yolk separator, and then stored at −20°C for subsequent research. After feeding, the laying hens were euthanized by cervical dislocation, and the hypothalamus, pituitary gland, liver, pancreas, duodenum, jejunum, ileum, cecum, gizzard, and abdominal adipose tissues were immediately collected, a total of 10 tissue samples. All intestinal segments and gizzard tissues were treated with content removal, and all intestinal segments, gizzard, liver, and abdominal fat tissues were weighed and recorded with a microbalance. Complete tissue samples were available for all 248 study subjects. Finally, all tissue samples were frozen in liquid nitrogen and stored at −80°C for subsequent studies.

### Determination of biochemical indicators

To gain a deeper understanding of the lipid content and related indicators in egg yolk, liver, and abdominal fat, we conducted biochemical index determination. The measurement indicators included egg yolk dry matter (**EYDMC**), egg yolk triglycerides (**EYTG**), egg yolk free fatty acid content (**EYFFA**), liver dry matter (**LDMC**), liver triglycerides (**LTG**), liver free fatty acid content (**LFFA**), liver cholesterol content (**LTC**), abdominal fat dry matter (**AFDMC**), abdominal fat triglyceride (**AFTG**), and abdominal fat-free fatty acid content (**AFFFA**).

Among them, the dry matter content (**DMC**) of these three types of tissue samples (egg yolk, liver, and abdominal fat) was determined according to the solid sample drying method of China National Standard (GB 5009.3-2016). The specific operation was to accurately weigh a small amount of samples (0.1 to 3.0 g) using an electronic balance, place them in a weighing flask, and then put them in an oven to dry until constant weight (105°C, 6 h). Finally, the poor mass before and after drying was weighed as the moisture content value in the tissue sample to calculate the dry matter content of the sample.

The triglyceride content of the three tissue samples was determined according to the principle of enzyme-linked immunosorbent assay (**ELISA**). Sample processing involves accurately weighing the tissue sample (0.1-0.2 g) into a 2 mL tube. A nine-fold volume of homogenization medium (non-aqueous ethanol) and three grinding beads (one 3 mm and two 1 mm) are added. The mixture is homogenized at 75 Hz for 6 minutes in an ice-water bath, then centrifuged at 2500 rpm for 10 minutes to collect the supernatant. For the assay procedure, 250 μL of working solution is added to blank, standard, and sample wells. Then, 2.5 μL of distilled water (blank), calibrator (standard), or supernatant (sample) is pipetted into the respective wells. After gentle mixing, the plate is incubated at 37°C for 10 minutes, and absorbance is measured at 500 nm using a SpectraMax i3x microplate reader. For specific operational steps, please refer to the triglyceride (**TG**) kit (A110-1-1, Nanjing Jiancheng Biological Engineering Institute) Operating Instructions. Free fatty acid and liver cholesterol contents were obtained from the Biotechnology Company (Nanjing Jiancheng Bioengineering Research Institute). The determination process was performed according to the operating instructions of the free fatty acid (**FFA**) kit (A042-2-1, enzyme method, microplate method) and total cholesterol (**TC**) kit (AKFA002M, CHOD-PAP method). In addition, free fatty acid (FFA) determination procedure involves precisely weighing tissue samples were homogenized with ice-cold homogenization buffer (60 % ethanol) at a 1:10 (w/v) ratio (minimum 300 μL for samples < 0.03 g) using a high-speed grinder. The homogenate was centrifuged at 2500 × *g* for 10 min, and the supernatant was diluted 4-fold for subsequent analysis. Each tissue sample were measured twice in parallel.

### Estimation of residual feed intake (E-RFI) at 70 to 100 weeks of age

The calculation of metabolic body weight (**MBW**) and RFI for the stage of 70 to 100 weeks followed the method of Yan et al.(Yan et al., 2019). Estimation was conducted using the lm() function of R software (version 4.2.2). The specific calculation formula for the RFI is as follows:

$$RFI=DFI-\left(b_{0}+b_{1}\times MBW+b_{2}\times DEM+b_{3}\times DBWG\right)$$

Among them, RFI represents the estimated value (**E-RFI**) of the remaining feed intake from 70 to 100 weeks of age. *b_0_* is the intercept, and *b_1_, b_2_*, and *b_3_* are partial regression coefficients. MBW is the metabolic body weight, which is calculated by taking the 0.75th power of the average weight of the 70th and 100th week of age; DFI, DEM, and DBWG are the daily average feed intake, daily average egg production, and daily average body weight gain from 70 to 100 weeks of age, respectively.

### RNA extraction and sequencing

We conducted transcriptome sequencing of seven tissues, including the hypothalamus (*n* = 19), pituitary gland (*n* = 20), liver (*n* = 20), pancreas (*n* = 20), duodenum (*n* = 20), ileum (*n* = 20), and cecum (*n* = 20). The specific operation steps are as follows.

### RNA extraction, library construction and sequencing

The total RNA quantity and purity were analysis of Bioanalyzer 2100 and RNA 6000 Nano LabChip Kit (Agilent, CA, USA, 5067-1511), high-quality RNA samples with RIN number > 7.0 were used to construct sequencing library. After total RNA was extracted, mRNA was purified from total RNA (5ug) using Dynabeads Oligo (dT) (Thermo Fisher, CA, USA) with two rounds of purification. Following purification, the mRNA was fragmented into short fragments using divalent cations under elevated temperature (Magnesium RNA Fragmentation Module (NEB, cat.e6150, USA) under 94°C 5-7 min). Then the cleaved RNA fragments were reverse-transcribed to create the cDNA by SuperScript™ II Reverse Transcriptase (Invitrogen, cat. 1896649, USA), which were next used to synthesise U-labeled second-stranded DNAs with E. coli DNA polymerase I (NEB, cat.m0209, USA), RNase H (NEB, cat.m0297, USA) and dUTP Solution (Thermo Fisher, cat.R0133, USA). An A-base was then added to the blunt ends of each strand, preparing them for ligation to the indexed adapters. Each adapter contained a T-base overhang for ligating the adapter to the A-tailed fragmented DNA. Dual-index adapters were ligated to the fragments, and size selection was performed with AMPureXP beads. After the heat-labile UDG enzyme (NEB, cat.m0280, USA) treatment of the U-labeled second-stranded DNAs, the ligated products were amplified with PCR by the following conditions: initial denaturation at 95°C for 3 min; 8 cycles of denaturation at 98°C for 15 sec, annealing at 60°C for 15 sec, and extension at 72°C for 30 sec; and then final extension at 72°C for 5 min. The average insert size for the final cDNA librarys were 300 ± 50 bp. At last, we performed the 2 × 150 bp paired-end sequencing (PE150) on an Illumina Novaseq™ 6000 (LC-Bio Technology CO., Ltd., Hangzhou, China) following the vendor's recommended prot.

### Sequencing data processing

To get high quality clean reads, reads were further filtered by Cutadapt (https://cutadapt.readthedocs.io/en/stable/, version: cutadapt-1.9). The parameters were as follows: 1) removing reads containing adapters; 2) removing reads containing polyA and polyG; 3) removing reads containing more than 5 % of unknown nucleotides (N); 4) removing low quality reads containing more than 20 % of low quality (Q-value ≤ 20) bases. Then sequence quality was verified using FastQC (http://www.bioinformatics.babraham.ac.uk/projects/fastqc/, 0.11.9). including the Q20, Q30 and GC-content of the clean data(Kechin et al., 2017; Thompson et al., 2020). We aligned reads of all samples to the chicken reference genome Gallus gallus using HISAT2 (https://daehwankimlab.github.io/hisat2/, version: hisat2-2.0.4) package, which initially remove a portion of the reads based on quality information accompanying each read and then maps the reads to the reference genome. HISAT2 allows multiple alignments per read (up to 20 by default) and a maximum of two mismatch when mapping the reads to the reference(Kim et al., 2015, 2019; Pertea et al., 2016). The mapped reads of each sample were assembled using StringTie (http://ccb.jhu.edu/software/stringtie/, version: stringtie-1.3.4d) with default parameters. Then, all transcriptomes from all samples were merged to reconstruct a comprehensive transcriptome using gffcompare software (http://ccb.jhu.edu/software/stringtie/gffcompare.shtml, version: gffcompare-0.9.8). After the final transcriptome was generated, StringTie and ballgown (http://www.bioconductor.org/packages/release/bioc/html/ballgown.html) were used to estimate the expression levels of all transcripts and determine the expression abundance of mRNAs by calculating **FPKM** (fragment per kilobase of transcript per million mapped reads) value(Kovaka et al., 2019; Pertea et al., 2015, 2016).

### Statistical method

#### Data preprocessing

All indicators were descriptively analyzed using R software (version 4.2.2), including the mean (**Mean**), maximum (**Maximum**), minimum (**Minimum**), standard deviation (**SD**), and coefficient of variation (**CV**). Prior to estimating genetic parameters, the normality of all traits was assessed using the Shapiro–Wilk test within this software. For traits that did not conform to a normal distribution, the GenABEL package of the R software was employed for normal transformation. To identify genes significantly associated with feed efficiency, we selected 20 individuals with high and low E-RFI values from 248 experimental individuals (individuals were selected from the upper and lower 20 % percentiles of both metrics) and divided them into four groups according to the number of eggs produced from 70 to 100 weeks of age (EN_70-100__w_) for further analysis. Duncan’s test was used in SPSS 27.0 to evaluate the significance of all index differences among grouped individuals. The results were expressed as “mean ± standard deviation”, and the significance threshold was *P* < 0.05. The significance level was indicated by letters at the end of the results.

#### Phenotypic correlation and genetic parameter estimation

Pearson correlation analysis was performed for all traits using the R software package for phenotypic correlation. To further elucidate the genetic links between traits, we used GCTA 1.94.1 software for SNP-based heritability (***h^2^***) and genetic correlation estimates. GCTA software is an effective tool for analyzing genome-wide complex traits, as proposed by Yang et al. The software can estimate the variance components that genome-wide SNP markers can explain, to estimate the heritability and genetic correlation of traits(Yang et al., 2011). The constrained maximum likelihood method (**GREML**) is used to estimate variance components. The variance component model estimated to be interpreted by genome-wide SNP markers is:

$$y=X\beta +g+\varepsilon ,V=A_{g}{\sigma^{2}}_{g}+I{\sigma^{2}}_{\varepsilon }$$

where *y* is the phenotype value; *β* is the fixed effect; *X* is the association matrix of *β; g* is the genetic effect of the individual; *A _g_* is the inter-individual kinship matrix (**GRM**) calculated by GCTA; *σ^2^*_g_ is the addition of SNP variance of sexual effect; *I* is the unit matrix; *σ^2^_ε_* is the residual.

#### Differential expression analysis

The resulting gene expression data were combined with the phenotypic data, and the principal component analysis graph (**PCA**) was plotted using the factoextra package of R software. Through dimensionality reduction, we can identify the grouping and clustering of samples more intuitively. EdgeR is a bioinformatic software package used for RNA-Seq data analysis. It provides a standardized analysis process, enhances the repeatability and transparency of research, and provides crucial information for studying changes in gene expression under different conditions and for understanding biological processes(Robinson et al., 2010). Therefore, we used the edgeR package to perform differential expression analysis of the gene expression data of the low-E-RFI and high-E-RFI groups. We corrected counts based on Counts per million (**CPM**) value, filtered out genes expressed in only a very small number of samples, and used the corrected *P* value < 0.05 (FDR < 0.05) and |log_2_FC| ≥ 1 as screening difference standards for expressing genes. Meanwhile, volcano maps were plotted using the ggplot2 package of the R software to visualize the analysis results, which facilitates the understanding of changes in gene expression. Finally, we used the online platform (http://vip.sangerbox.com) to draw Upset charts to comprehensively analyze the distribution characteristics of differentially expressed genes in different tissues.

#### Functional enrichment analysis

Differentially expressed genes (**DEGs**) from seven tissues were subjected to Gene Ontology (GO) classification(Ashburner et al., 2000) and Kyoto Encyclopedia of Genes and Genomes (KEGG) pathway enrichment analysis(Kanehisa, 2000) using the DAVID (version 6.8) online analysis tool with species-specific annotation (Gallus gallus)(Huang et al., 2009; Sherman et al., 2022). GO terms were categorized into three ontologies: biological processes (**BP**), cellular components (**CC**), and molecular functions (**MF**). Significant enrichment thresholds were set at *P* < 0.05 for both GO terms and KEGG pathways. The bioinformatics platform (http://www.bioinformatics.com.cn) generated visualization graphics (bubble plots/histograms) to display the enrichment patterns.

#### Weighted Gene Co-expression Network Analysis

Weighted Gene Co-expression Network Analysis (WGCNA), as a method for analyzing the correlation of gene expressions in samples, has become an important tool in bioinformatics analysis(Langfelder and Horvath, 2008a). We used the WGCNA package of the R software to systematically analyze the co-expression modules related to E-RFI in the hypothalamus, pituitary, liver, pancreas, duodenum, ileum, and cecum tissues. The specific process was as follows: First, based on gene expression variance screening (excluding the 20 % of genes with the lowest variance), a scale-free co-expression network was constructed. Second, the Pearson's correlation matrices and average linkage method were both perfomed for all pair-wise Genes, Then, a weighted adjacency matrix was constructed using a power funcion A _mn=|C_mnl|^β (C_mn = Pearson's corelation between Gene_m and Gene_n; A_mn=adjacency between Gene m and Gene n), an appropriate “soft threshold” was determined by the soft threshold function (pickSoftThreshold, optimized in the 1-30 interval). Soft thresholds setting values: hypothalamus (1), pituitary (28), liver (20), Pancreas (8), duodenum (5), ileum (6), and cecum (16). After choosing the power, the adjacency was transformed into a topological overlap matrix (TOM), which could measure the network connectivity of a Gene defined as the sum of its adiacency with all other Genes for network Gene ration, and the coresponding dissimilarity (1-TOM) was calculated. To classify Genes with similar expression profiles into Gene modules, average linkage hierarchical clustering was conducted according to the TOM-based dissimlarity measure with a minimum size (Gene aroup) of 30 for the Genes dendrogram, set the sensitivity to: 3. To further analyze the module, we calculated the dissimilarity of module eigen Genes, chose a cut line for module dendrogram and merged some module (merging threshold *r* ≥ 0.25). Then, use the “Module Eigengenes” function to calculate the module eigengenes. Based on the module eigengenes, use the “corPvalueStudent” function to perform correlation analysis between the module and E-RFI for each tissue. It is worth noting that based on the sample size limitation (*n* = 20), we use the analysis strategy of uncorrected p-value (< 0.05) and correlation coefficient to screen the significant modules. Finally, GS and MM were obtained by calculating the correlation between gene expression and the correlation between the module eigenvector and gene expression, respectively. Set screening thresholds of |MM| > 0.8 and |GS| > 0.1 to extract genes in significant co-expression modules (Langfelder and Horvath, 2008b; Tang et al., 2018).

#### Protein-protein interaction (PPI) network analysis

To analyze the mechanism of collaborative regulation of genes in inter-tissue modules, we constructed a protein interaction network based on the STRING 11 database (Szklarczyk et al., 2023). The specific parameters are as follows: “Gallus gallus” is selected as the research object, the network type is the full STRING network, a medium confidence threshold (0.400) is set to screen interaction relationships, and unconnected nodes are hidden to optimize network visualization. The analysis parameters were set with term similarity merging disabled (no merging of rows), a maximum FDR threshold of ≤ 0.05, minimum signal and strength thresholds of ≥ 0.01 each, and a minimum network count requirement of ≥ 2 to ensure rigorous yet biologically relevant network analysis.

#### Hub gene screening

To identify hub genes associated with E-RFI, we analyzed the node interaction results obtained from the protein-protein interaction network analysis in section 1.5.6 using the Analysis Network function in Cytoscape software (version 3.10.3). The core genes were ranked and filtered based on the degree values of the network. Specifically, genes with a degree value ≥ 28 were selected for the liver, those with a degree value ≥ 6 were selected for the duodenum, and those with a degree value ≥ 36 were selected for the cecum.

## Results

### Phenotype descriptive statistics

The daily feed intake, body weight, and egg weight were measured for each laying hen during two age periods (70 to 71 weeks and 99 to 100 weeks), while the total egg production was recorded continuously from 70 to 100 weeks. The estimated residual feed intake (E-RFI) at this stage was calculated to evaluate feed efficiency (see Materials and methods). Concurrently, key lipid biochemical parameters were determined to characterize lipid metabolism patterns. The descriptive statistics for the core traits are summarized in Table 1, with comprehensive raw data provided in Table S1.

**Table 1:** Descriptive statistics of production and lipid indicators at ages 70 to 100 weeks.

Traits	Mean	Maximum	Minimum	SD	CV (%)
E-RFI	0	29.46	−43.81	10.99	—
TEN100	479.25	569	175	63.46	13.24
EN_70-100w_	146.61	213	1	42.92	29.28
AEW_70-100__w_ (g)	59.07	73	43.5	4.01	6.79
AYW_70-100__w_ (g)	16.51	21.6	13.6	1.39	8.40
DFI_70-100__w_ (g/d)	107.98	140.02	63.34	13.13	12.16
DEM_70-100__w_ (g/d)	41.86	60.07	3.94	11	26.27
DBWG_70-100__w_ (g/d)	−0.04	2.43	−2.86	0.86	−2355.22
BW70 (g)	2079.7	2970	1490	218.33	10.50
BW100 (g)	2071.54	3185	1350	251.98	12.16
DFI_70-71__w_ (g/d)	110.08	137.11	60.97	12.84	11.66
DFI_99-100__w_ (g/d)	106.16	163.4	17.17	18.51	17.44
DEM_70-71__w_ (g/d)	44.44	65.49	0	13.19	29.68
DEM_99-100__w_ (g/d)	34.11	65.23	0	18.14	53.17
DBWG_70-71__w_ (g/d)	−4.63	14.53	−30.51	6.02	−129.99
DBWG_99-100__w_ (g/d)	1.25	25.38	−31.15	6.87	550.26
EW100 (g)	61.61	77.5	45.5	5.64	9.16
YW100 (g)	17.32	24.1	11.3	1.87	10.81
LW (g)	35.9	66.8	22.29	8.64	24.05
AFW (g)	90.17	290.8	4.7	43.62	48.38
EYDMC (%)	51.34 %	55.58 %	34.39 %	0.03	5.23
EYTG (μmol/g)	44.81	58.48	29.57	6.05	13.49
EYFFA (μmol/g)	2.07	3.92	1.11	0.54	26.27
LDMC (%)	39.79 %	71.97 %	23.17 %	0.08	21.02
LTG (μmol/g)	30.03	72.72	6.71	15.05	50.13
LFFA (μmol/g)	23.11	40.73	12.7	5.96	25.78
LTC (μmol/g)	4.37	12.12	2.09	1.56	35.58
AFDMC (%)	96.62 %	99.30 %	86.92 %	0.02	2.14
AFTG (μmol/g)	147.79	220.88	82.52	36.5	24.7
AFFFA (μmol/g)	2.32	6.31	0.37	0.91	39.03

Note: SD = standard deviation; CV = coefficient of variation; E-RFI = estimated residual feed intake at 70 to 100 weeks of age; TEN100 = total number of eggs laid in the first 100 weeks of age; EN_70-100__w_ = number of eggs laid at 70 to 100 weeks of age; AEW_70-100__w_ = average egg weight at 70 to 100 weeks of age; AYW_70-100__w_ = average egg yolk weight at 70 to 100 weeks of age; DFI_70-100__w_ = average daily feed intake at 70 to 100 weeks of age; DEM_70-100__w_ = average daily egg mass at 70 to 100 weeks of age; DBWG_70-100__w_ = average daily body weight gain at 70 to 100 weeks of age; BW70 and BW100 = body weight at 70th and 100th weeks of age; DFI_70-71__w_ and DFI_99-100__w_ = average daily feed intake at 70 to 71 weeks and 99 to 100 weeks of age; DEM_70-71__w_ and DEM_99-100__w_ = average daily egg mass at 70 to 71 weeks and 99 to 100 weeks of age; DBWG_70-71__w_ and DBWG_99-100__w_ = average daily body weight gain at 70 to 71 weeks and 99 to 100 weeks of age. EW100 and YW100 = egg weight and egg yolk weight at the 100th week of age; LW and AFW = liver weight and abdominal fat weight at the 100th week of age; EYDMC = the dry matter content of egg yolk at the 100th week of age; EYTG = triglyceride content of egg yolk at the 100th week of age; EYFFA = free fatty acid content of egg yolk at the 100th week of age; LDMC = liver dry matter content at the 100th week of age; LTG = liver triglyceride content at the 100th week of age; LFFA = liver free fatty acid content at the 100th week of age; LTC = liver cholesterol content at the 100th week of age; AFDMC = abdominal fat dry matter content at the 100th week of age; AFTG = abdominal fat triglyceride content at the 100th week of age; AFFFA = abdominal fat free fatty acid content at the 100th week of age.

As shown in the table, DFI_70-100__w_ (CV = 12.16 %), DEM_70-100__w_ (CV = 26.27 %), and DBWG_70-100__w_ (CV = −2355.22 %) exhibited moderate to high levels of variation, indicating significant genetic heterogeneity within the population. This reflects substantial differences in egg production and feed utilization efficiency among individuals, leading to asynchronous changes in egg production and body weight. Furthermore, the lipid biochemical indicators of the yolk (CV = 5.23-26.27 %), liver (CV = 21.02-50.13 %), and abdominal fat (CV = 2.14-39.03 %) showed wide variability, suggesting divergent energy allocation strategies among individuals.

### Genetic parameter estimation of E-RFI and related traits

To further elucidate the genetic characteristics of these traits, we assessed the genetic parameters for E-RFI and Related Traits (Table 2, details in Table S2). Heritability (*h²*) analysis revealed moderate heritability for E-RFI (0.45), aligning with conventional RFI estimates. The heritability of average daily feed intake (0.53) and average daily body weight gain (0.22) at 99 to 100 weeks of age was higher than that at 70 to 71 weeks of age, whereas the heritability of average daily egg mass (0.15) was lower than that at 70 to 71 weeks of age. This indicates that genetic selection for feed efficiency and growth rate should be prioritized during the late laying phase, whereas genetic improvement of egg production should focus on earlier stages. Furthermore, lipid metabolism traits, including dry matter content (DMC, 0.17-0.39), triglycerides (TG, 0.01-0.19), free fatty acids (FFA, 0.15-0.17), and total cholesterol (TC, 0.08), exhibited moderate to low heritability, whereas nutrient metabolism-related traits generally demonstrated moderate to high heritability (0.36-0.60), highlighting that lipid metabolism traits are strongly influenced by environmental or management factors (low heritability) and thus require optimization through nutritional regulation, while their potential for genetic improvement remains limited.

**Table 2:** Phenotypic and genetic correlation analysis of the main traits.

Traits	EN_70-100w_	AEW_70-100w_	AYW_70-100w_	EW100	YW100	LW	AFW	EYDMC	EYTG	EYFFA	LDMC	LTG	LFFA	LTC	AFDMC	AFTG	AFFFA	E-RFI
EN_70-100w_	0.37 (0.18)	−0.48	−0.42	−0.72	−0.66	0.32	0	0.19	0.46	−0.6	−0.02	0.28	−0.17	−0.02	0.15	1	−0.36	0.22
AEW_70-100w_	−0.20⁎⁎	0.64 (0.19)	1	1	1	−0.41	0.06	−0.04	−0.13	0.3	−0.06	−0.01	−0.19	0.66	0.26	−1	−0.45	0.11
AYW_70-100w_	−0.19⁎⁎	0.46⁎⁎	0.09 (0.15)	0.81	1	−1	−0.24	−0.61	1	0.15	−1	−0.54	−0.69	1	0.58	1	−0.13	0.42
EW100	−0.22⁎⁎	0.80⁎⁎	0.41⁎⁎	0.47 (0.26)	1	−0.52	0.18	−0.57	1	0.59	0.26	−0.29	−0.21	0.54	0.22	1	−0.32	−0.33
YW100	−0.24⁎⁎	0.45⁎⁎	0.80⁎⁎	0.56⁎⁎	0.23 (0.23)	−1	−0.3	−0.57	−0.61	0.99	−0.46	−0.7	−0.55	−0.27	−0.26	−1	0.11	0.25
LW	−0.1	−0.09	0.18⁎⁎	0.06	0.20⁎⁎	0.21 (0.15)	0.29	0.13	−1	−0.66	1	1	0.72	1	0.66	1	−0.4	0.01
AFW	−0.03	0.09	0.26⁎⁎	0.17*	0.25⁎⁎	0.41⁎⁎	0.59 (0.17)	−0.05	1	0.6	0.57	0.51	0.46	1	0.9	−1	−0.32	−0.6
EYDMC	0.01	0.13	0.21⁎⁎	0.14	0.16*	0.19*	0.19⁎⁎	0.20 (0.21)	1	0.51	0.21	0.37	0.23	1	−0.44	−1	−1	0.58
EYTG	0.01	−0.03	0.05	0.05	0.1	0.14	0.02	0	0.11 (0.18)	0.94	−0.29	0.58	0.2	1	0.34	−1	0.91	1
EYFFA	−0.22⁎⁎	−0.04	−0.05	−0.04	0.02	−0.04	0.07	−0.12	0.03	0.15 (0.22)	0.04	−0.64	0.18	0.81	0.08	−1	0.27	−0.35
LDMC	0.01	0.08	0.13*	0.08	0.13	0.43⁎⁎	0.29⁎⁎	0.08	−0.03	0.07	0.17 (0.14)	1	1	1	1	−1	0.81	−0.2
LTG	−0.03	0.07	0.14*	0.1	0.15	0.41⁎⁎	0.34⁎⁎	0.14	−0.01	0.04	0.24⁎⁎	0.19 (0.15)	0.84	1	0.81	−1	−1	0.03
LFFA	0.01	−0.04	0.08	0.01	0.03	0.33⁎⁎	0.32⁎⁎	0.04	−0.06	0.08	0.28⁎⁎	0.46⁎⁎	0.17 (0.16)	1	0.49	1	−1	−0.28
LTC	−0.08	0.08	0.22⁎⁎	0.11	0.18*	0.22⁎⁎	0.27⁎⁎	0.08	−0.01	0.03	0.37⁎⁎	0.08	0.06	0.08 (0.13)	1	1	−0.31	0.39
AFDMC	0.08	0.07	0.17⁎⁎	0.14	0.15	0.18⁎⁎	0.59⁎⁎	0.08	−0.03	0.04	0.23⁎⁎	0.29⁎⁎	0.32⁎⁎	0.19⁎⁎	0.39 (0.17)	−1	−0.63	−0.48
AFTG	−0.02	0	0.06	−0.02	0.08	0	0.06	−0.04	−0.11	0.08	0.09	0.1	0.11	0.02	0.22⁎⁎	0.01 (0.11)	1	1
AFFFA	−0.01	−0.07	−0.12	−0.05	−0.13	−0.24⁎⁎	−0.29⁎⁎	−0.06	0.03	0.02	−0.16*	−0.22⁎⁎	−0.04	−0.08	−0.19⁎⁎	0.01	0.15 (0.14)	−0.12
E-RFI	0.03	0.01	0.03	0.08	0.15*	0.27⁎⁎	−0.19⁎⁎	0.01	0.03	−0.11	0.05	−0.06	−0.01	−0.02	−0.14*	0.07	−0.05	0.45 (0.19)

Note: Heritability is listed on the diagonal, the values in parentheses represent the standard errors; genetic correlation coefficients are listed above the diagonal, and phenotypic correlation coefficients are listed below. In phenotypic correlations:.

⁎⁎ indicates significance at the 0.01 level (two-tailed).

⁎ indicates significance at the 0.05 level (two-tailed). Letter annotations are shown in Table 1.

Genetic correlation analysis revealed that E-RFI exhibited strong phenotypic and genetic positive correlations with daily feed intake during the 70 to 100 weeks of age (DFI_70-100__w_), but weaker associations with egg number (EN_70-100w_: 0.22) and egg weight (AEW_70-100w_: 0.11). Notably, E-RFI showed a high negative genetic correlation with body weight gain during this period (DBWG_70-100w_: −0.97), indicating an antagonistic energy allocation between growth and egg production. In lipid metabolism, E-RFI displayed moderate negative genetic correlations with egg weight(**EW100**) and EYFFA at 100 weeks of age, and moderate to high positive correlations with AYW_70-100__w_, EYDMC, and yolk weight at 100 weeks of age (**YW100**). These results suggest that improved feed efficiency may enhance egg weight at the cost of reduced yolk nutrient reserves, reflecting a genetic trade-off in energy allocation between egg production and lipid metabolism. Additionally, E-RFI exhibited low-moderate positive correlations with LW and LTC, but moderate negative correlations with LDMC and LFFA, highlighting functional divergence in hepatic lipid metabolism. Furthermore, E-RFI showed moderate to high negative correlations with AFW and AFDMC, and a weak negative correlation with AFFFA. This pattern aligns with lipid composition changes in egg yolks, suggesting that the liver coordinates egg production demands and energy homeostasis through different lipid synthesis pathways, thereby maintaining a dynamic balance between fat deposition and follicular development.

### Grouping by RFI and egg production

From a flock of 248 experimental chickens, four comparative groups were constructed based on dual traits (see Table 3, details in Table S3): E-RFI and egg number from 70 to 100 weeks of age (EN_70-100__w_). Group 1 and 2 exhibited excellent feed efficiency (Group 1: −11.96 ± 3.45; Group 2: −13.37 ± 2.45), but differed significantly in egg production (Group 1: 184.8 ± 7.58; Group 2: 94.7 ± 38.05) (*P* < 0.05). Similarly, Groups 3 and 4 displayed poor feed efficiency (Group 3:14.66 ± 3.49; Group 4:14.85 ± 3.23), while Group 3 maintained high egg production (184.7 ± 6.82), comparable to Group 1. This experimental design, particularly the contrast between Group 1 (high efficiency, high production) and Group 3 (low efficiency, high production), effectively isolated the genetic effects of egg production traits by controlling for egg production. By decoupling RFI from confounding production traits, this approach overcomes the limitations of traditional single-trait feed efficiency grouping, significantly improving the accuracy and reliability of the molecular analyses of RFI regulation.

**Table 3:** Comparison of main production indicators of four groups of individuals.

Traits	L-ERFI		H-ERFI	
	Group 1 (*n* = 10, High egg production)	Group 2 (*n* = 10, Low egg production)	Group 3 (*n* = 10, High egg production)	Group 4 (*n* = 10, Low egg production)
E-RFI	−11.96 ± 3.45^b^	−13.37 ± 2.45^b^	14.66 ± 3.49^a^	14.85 ± 3.23^a^
EN_70-100w_	184.8 ± 7.58^a^	94.7 ± 38.05^b^	184.7 ± 6.82^a^	113.9 ± 21.52^b^
DFI_70-100__w_ (g/d)	99.63 ± 4.12^c^	88.92 ± 4.83^d^	127.56 ± 7.13^a^	117.94 ± 3.02^b^
DBWG_70-100__w_ (g/d)	−0.04 ± 0.65^ab^	0.62 ± 0.92^a^	−0.43 ± 0.69^b^	0.19 ± 0.64^ab^
LW (g)	29.45 ± 2.38^b^	34.21 ± 9^ab^	35.26 ± 7.01^ab^	41.32 ± 14.55^a^
AFW (g)	93.63 ± 33.33^b^	135.69 ± 44.83^a^	75.4 ± 40.81^b^	67.09 ± 27.2^b^
EYDMC (%)	51.05 % ± 2.36 %^a^	52.18 % ± 3.25 %^a^	50.50 % ± 2.80 %^a^	50.92 % ± 2.11 %^a^
EYTG (μmol/g)	46.38 ± 5.43^a^	43.65 ± 1.97^a^	46.1 ± 6.75^a^	44.14 ± 3.52^a^
EYFFA (μmol/g)	2.29 ± 0.41^ab^	2.45 ± 0.16^a^	1.78 ± 0.32^b^	2.1 ± 0.62^ab^
LDMC (%)	39.33 % ± 12.62 %^a^	42.57 % ± 6.73 %^a^	35.76 % ± 2.90 %^a^	39.54 % ± 7.31 %^a^
LTG (μmol/g)	22.66 ± 11.9^a^	32.39 ± 16.93^a^	32.73 ± 18.86^a^	32.18 ± 20.12^a^
LFFA (μmol/g)	22.41 ± 5.27^a^	23.1 ± 6.12^a^	24.19 ± 6.64^a^	20.06 ± 4.5^a^
LTC (μmol/g)	4.16 ± 0.9^a^	4.8 ± 1.68^a^	3.68 ± 0.58^a^	4.27 ± 1.24^a^
AFDMC (%)	97.27 % ± 2.48 %^a^	96.47 % ± 1.97 %^a^	96.71 % ± 1.03 %^a^	96.50 % ± 1.52 %^a^
AFTG (μmol/g)	159.49 ± 39.77^a^	124.6 ± 27.72^b^	143.46 ± 32.83^ab^	135.81 ± 35.74^ab^
AFFFA (μmol/g)	2.34 ± 1.04^a^	2.23 ± 0.73^a^	1.98 ± 1.09^a^	1.84 ± 0.87^a^

Note: l-ERFI = low residual feed intake group; H-ERFI = high residual feed intake group; E-RFI = estimated residual feed intake at 70 to 100 weeks of age. Letter annotations are shown in Table 1. a-c means values with no common superscripts within each row differ significantly (*P* < 0.05) when tested with Duncan’s test. Values are expressed as “mean ± standard deviation”.

The phenotypic trends of daily feed intake, egg production, and body weight gain across the four groups from 70 to 100 weeks of age were consistent with the findings of the genetic correlation analysis. Notably, the differences observed in EYFFA among the groups were partially consistent with the negative genetic correlation between E-RFI and EYFFA, further validating the genetic regulatory link between E-RFI and lipid metabolism traits.

### Differential gene screening and functional enrichment analysis

Based on the comparative analysis, Groups 1 and 3 displayed similarly high egg production performance, yet exhibited significant divergence in E-RFI, reflecting distinct nutrient utilization efficiencies. To investigate the underlying genetic mechanisms driving these E-RFI differences, we performed transcriptome profiling across seven key metabolic and regulatory tissues: hypothalamus, pituitary gland, liver, pancreas, duodenum, ileum, and cecum. Following quality control of the raw sequencing data, each sample yielded an average of 19.37 Million Reads, with an average annotation efficiency of 55.74 %. We detected the expression profiles of 16,382 protein-coding genes across all samples. Differential expression analysis was conducted using the edgeR package (R software) to identify statistically significant differentially expressed genes (DEGs) between groups (Table S4). The tissue-specific distribution of the DEGs is summarized in Table 4. Principal component analysis (PCA) and volcano plots (Figure S1) revealed limited sample dispersion in the PCA space between comparison groups. Across the seven tissues, Group 1 showed 32-103 down regulated and 75-1,236 up regulated genes compared to Group 3, respectively.

**Table 4:** Statistics of DEGs between Groups 1 and 3 in seven tissues.

Tissue	Down regulated	Up regulated	Total DEGs
Hypothalamus	40	162	202
Pituitary	89	75	164
Liver	103	163	266
Pancreas	83	1236	1319
Duodenum	32	99	131
Ileum	54	101	155
Cecum	42	243	285

Note: Down- and up-regulated genes refer to those significantly decreased or increased in Group 1 compared to Group 3 (|log₂FC| ≥ 1, FDR < 0.05).

**Fig. 1:**
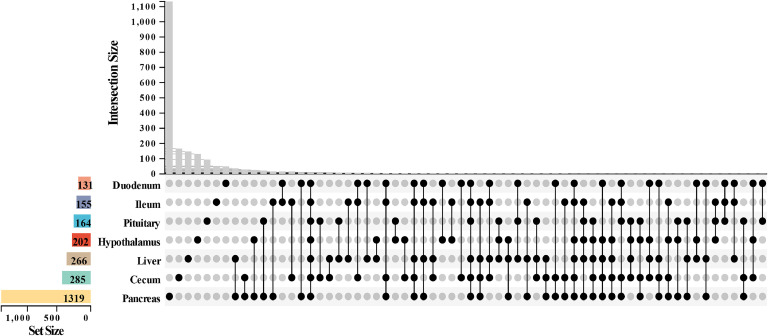
Upset diagram of differentially expressed genes in different organizations.

Upset analysis revealed hierarchical regulatory networks among tissue-specific DEGs (Fig. 1, Table S5). The hypothalamic-pituitary axis showed limited tissue-specific regulation, whereas pancreatic DEGs exhibited strong functional specificity (> 85 % specificity) with minimal co-regulation (small intersection size). As the metabolic hub, the liver coordinated a multi-tissue collaborative network (participating in 52.5 % of intersections) to efficiently integrate metabolic processes. Notably, we identified the largest co-regulated gene cluster between the pancreas and liver (*n* = 70), in contrast to the minimal core gene set shared by all seven tissues (*n* = 9). The intestinal segments (duodenum, ileum, and cecum) formed dynamic nutrient-processing networks through tissue-specific interactions to maintain systemic metabolic homeostasis.

The vertical axis represents the number of DEGs shared by each tissue combination. Each column on the horizontal axis (combination area) represents a tissue combination pattern. The black dots in the matrix indicate the tissues participating in this combination. The left Set Size axis labels the total number of DEGs that are independently contained in each tissue. The height of the column reflects the number of shared DEGs corresponding to the tissue combinations.

**Fig. 2:**
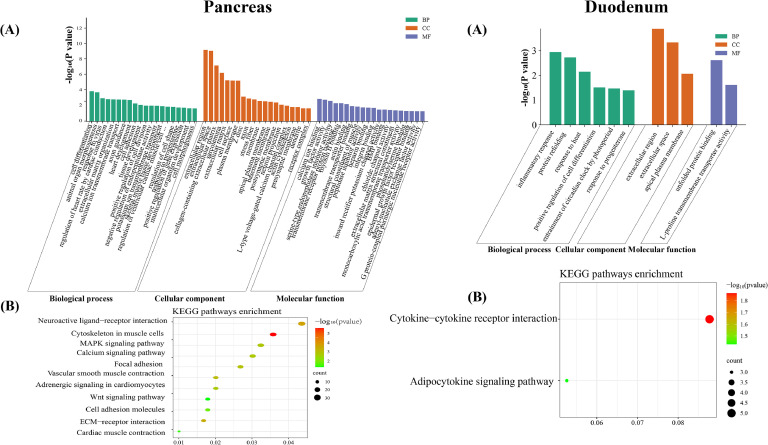
Partial organization GO column chart and KEGG bubble chart.

To functionally characterize DEGs, we performed comprehensive GO and KEGG enrichment analyses across all seven tissues (Tables S6 and S7), with annotation networks visualized in Fig. 2 (details in Figure S2). Our multi-tissue analysis revealed significant functional heterogeneity (*P* < 0.05), with 11-160 GO terms and 0-11 KEGG pathways enriched per tissue. The pancreas exhibited the most extensive functional spectrum, with the highest number of enriched GO terms and KEGG pathways. In contrast, the duodenum had the fewest enriched GO terms and the ileum showed no significant KEGG pathway enrichment. Cross-tissue KEGG analysis identified that 339 DEGs were significantly enriched in 21 pathways (*P* < 0.05, Table S8) governing reproductive development, immune modulation, energy metabolism balance, and tissue homeostasis. Tissue-specific analysis identified (1) duodenal adipocytokine signaling directly regulating lipid metabolism, and (2) cecal metabolic networks (glycine/serine/threonine metabolism, carbon metabolism, biosynthesis of amino acids, and tyrosine metabolism) as the central drivers of dietary carbon/nitrogen transformation and nutritional absorption efficiency. Notably, neuroactive ligand-receptor interactions (hypothalamic-pituitary-pancreas axis) and NOD-like receptor signaling (pituitary-liver axis) formed a core co-regulatory network with significant multi-tissue enrichment (*P* < 0.05).

**(A)** Map of significantly enriched GO entries in each tissue (*P* < 0.05). The green, orange and blue bars represent BP, CC and MF; respectively. **(B)** Diagram of significantly enriched KEGG pathways in each tissue (*P* < 0.05). For pancreatic tissue, only the top 20 items were displayed.

### WGCNA analysis and identification of co-expression modules

**Fig. 3:**
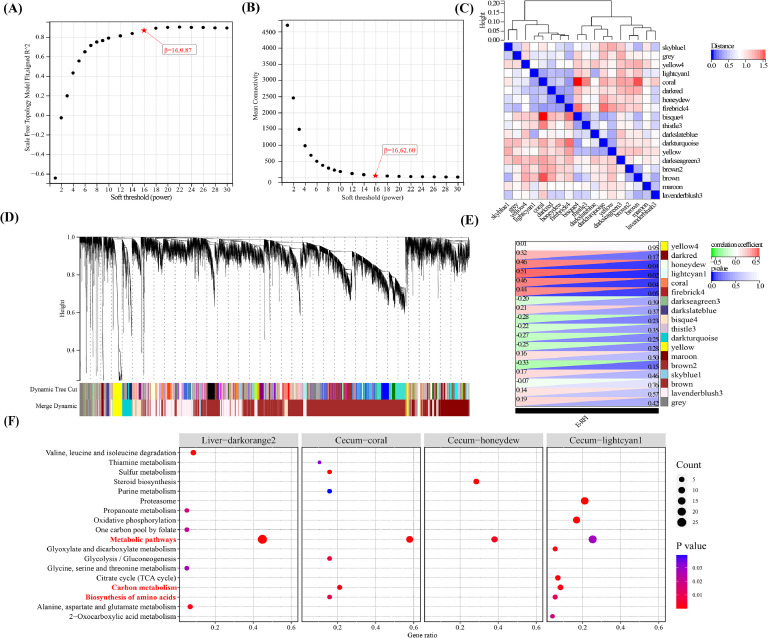
Weighted gene co-expression network analysis for some tissues.

To identify functional gene modules associated with E-RFI, we performed WGCNA analysis across seven tissues (hypothalamus, pituitary, liver, pancreas, duodenum, ileum, and cecum). The analysis identified tissue-specific co-expression modules (2, 15, 17, 6, 12, 19, and 18 modules per tissue; Figs.3 and S3). Correlation analysis revealed significant positive associations (*P* < 0.05) between E-RFI and the ​darkorange2 module in the liver (*r* = 0.55), ​sienna3 in duodenum (*r* = 0.56), ​coral (*r* = 0.46), ​honeydew (*r* = 0.46), and ​lightcyan1 (*r* = 0.51) modules in the cecum. Notably, these E-RFI-associated modules contained no overlapping genes and showed tissue specificity, as no significant modules were detected in the hypothalamus, pituitary, pancreas, or ileum.

Subsequently, threshold filtering (|MM| > 0.8, |GS| > 0.1) yielded core gene sets from significant modules: liver (darkorange2: 62 genes), duodenum (sienna3: 30 genes), and cecum (coral: 21, honeydew: 24, lightcyan1: 65 genes). Enrichment analysis of the E-RFI-associated gene modules revealed tissue-specific GO terms and KEGG pathways in the liver, duodenum, and cecum (Table S9). The liver and cecum exhibited 6 and 17 significantly enriched KEGG pathways (*P* < 0.05, Fig. 3F), respectively, whereas the duodenum showed only non-significant enrichment in the​ adipocytokine signaling pathway. Key metabolic pathway analysis demonstrated that genes from the liver ​(darkorange2 module) and cecum (​coral, ​honeydew, and ​lightcyan1 modules) were all significantly enriched in metabolic pathways, indicating a strong correlation between metabolic processes and feed efficiency. Notably, ​Carbon metabolism and ​biosynthesis of amino acids were shared between the cecal ​coral and ​lightcyan1 modules, with ​the lightcyan1 module uniquely enriched in ​oxidative phosphorylation—a pathway previously associated with feed intake and efficiency heterosis(Yuan et al., 2023). These findings align with the tissue-specific pathway enrichments, supporting the critical regulatory roles of carbon-nitrogen metabolic balance and cellular energy homeostasis in feed efficiency.

(A), (B), (C), (D), and (E) show the results of WGCNA analysis of cecum tissue. **(A)** Scale independence map. The abscissa represents the soft threshold and the ordinate represents the scale-free topological model fitting. β represents the value of the soft threshold. **(B)** Average connectivity map. The abscissa represents the soft threshold, and the ordinate represents the average connectivity. β represents the value of the soft threshold, and **(C)** module eigenvector clustering map. Both the abscissa and ordinate represent modules named with different colors; **(D)** gene clustering map; **(E)** module and phenotype correlation heat map. The color of the heat map represents the positive/negative Pearson correlation coefficient; **(F)** KEGG pathways significantly enriched by genes in the E-RFI-related modules for the liver and cecum.

### Cross-tissue protein-protein interaction (PPI) network analysis

**Fig. 4:**
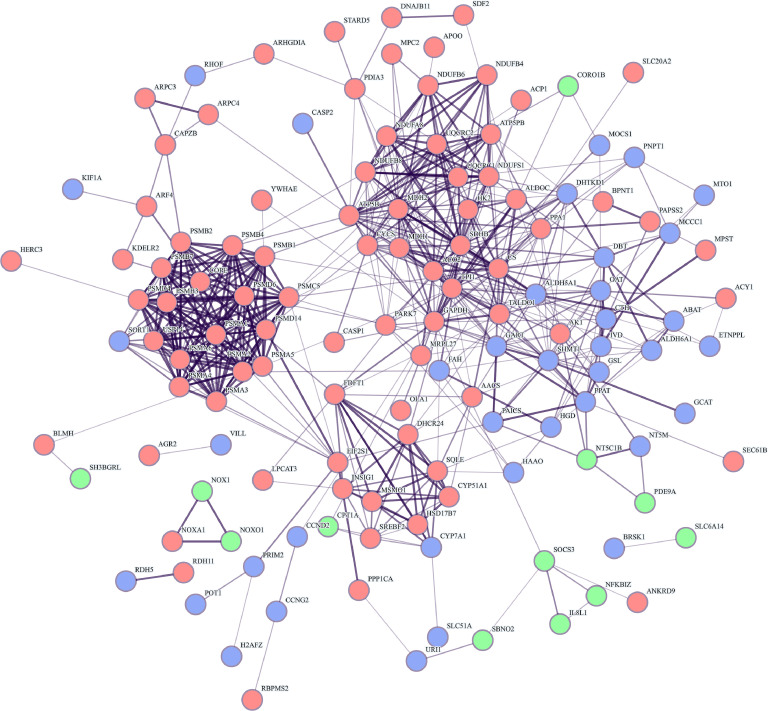
Protein interaction network of the union of significantly co-expressed module genes in liver, duodenum, and cecum tissues.

To elucidate the inter-tissue coordination mechanism of the liver, duodenum, and cecum in regulating feed efficiency, we focused on the five significantly E-RFI-related functional modules and investigated their coordinated regulatory pathways through protein-protein interaction (**PPI**) network analysis. STRING-based PPI network analysis of the merged gene sets revealed 176 protein nodes (derived from 183 annotated module genes) forming 556 interactions (Fig. 4), which demonstrated that the average node connectivity was 6.32, the average local clustering coefficient was 0.453, and the significance of protein interaction enrichment reached *P* < 1.0E-16. Notably, while no direct gene overlap was observed among the E-RFI-related modules across the three tissues, the PPI network uncovered significant cross-tissue functional associations Liver nodes exhibited high interconnectivity, forming the global network core, consistent with its role as the metabolic command center. Cecal nodes are primarily organized into local dense subnetworks, reflecting their functional modularity. Although fewer in number, duodenal nodes serve as critical cross-tissue connectors. These findings highlight a multi-tissue regulatory paradigm in which distinct yet interconnected functional architectures in the liver, duodenum, and cecum collectively govern feed efficiency.

Each node represents a single protein. The blue, green, and red nodes represent the proteins corresponding to the module genes significantly co-expressed in the liver, duodenum, and cecum, respectively. The lines between the nodes represent the associations between proteins. Line thickness indicates the strength of the data support. The unconnected nodes in the network were removed.

### Identification of hub genes via cytoscape

**Fig. 5:**
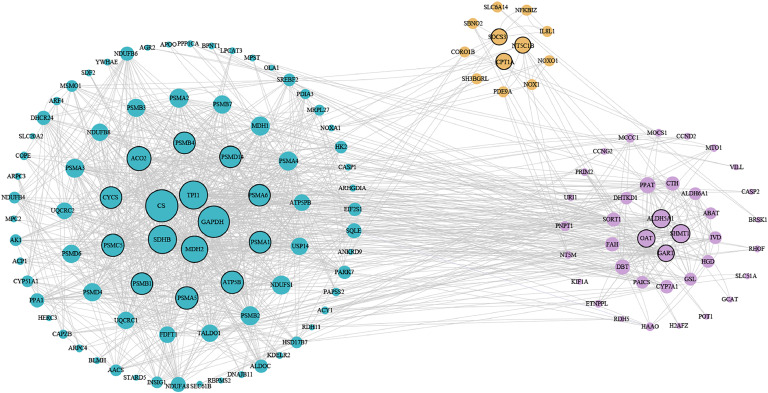
Cytoscape diagram of interacting genes.

To precisely identify candidate genes related to E-RFI in laying hens, we performed hub gene analysis using Cytoscape software in liver, duodenum, and cecal tissues based on the finding of protein-protein interaction network (Fig. 5). Our analysis revealed 4, 3, and 15 core hub genes in the liver, duodenum, and cecum, respectively (Table 5). Notably, the cecal PPI network exhibited a higher connectivity density than other tissues. However, genes within the honeydew module displayed significantly lower degrees of interaction than those in the coral and lightcyan1 modules. Consequently, candidate genes were selected exclusively from the latter two modules for further analyses. Functional annotation indicated that these hub genes are predominantly involved in energy metabolism, protein homeostasis regulation, immune signal transduction, and amino acid/lipid metabolism, suggesting that multi-pathway coordination of these biological processes plays a pivotal role in regulating growth performance and feed efficiency in laying hens.

Cytoscape graphs for the cecum, duodenum, and liver are represented in blue, orange, and purple, respectively. The black-outlined genes represent hub genes.

**Table 5:** Summary of hub genes related to feed efficiency.

Tissue	Gene name	Gene Function	Module
Liver	*ALDH5A1,SHMT1,OAT,GART*	Involved in amino acid and nucleotide metabolism	darkorange2
Duodenum	*SOCS3,NT5C1B,CPT1A*	Involved in immune and signal regulation, nucleotide and lipid metabolism	sienna3
Cecum	*CYCS,GAPDH*	Involved in energy metabolism and oxidative phosphorylation	coral
	*CS,SDHB,TPI1,MDH2,ACO2,PSMA5,ATP5B,PSMC5,PSMA1,PSMB4,PSMB1,PSMA6,PSMD14*	Involved in energy metabolism, oxidative phosphorylation, and protein homeostasis regulation	lightcyan1

## Discussion

Enhancing feed efficiency and reducing production costs in laying hens during prolonged laying cycles have become critical challenges in the poultry industry. Previous studies have demonstrated that artificial selection strategies effectively improve feed utilization efficiency in laying hens(Van Kaam et al., 1999; Zhou et al., 2023), establishing this trait as a key economic indicator for commercial chicken breeding(Urgessa and Woldesemayat, 2023). Deciphering the genetic regulatory mechanisms will advance the precise breeding of high-efficiency, high-yield individuals.

Notably, current research on RFI predominantly focuses on whole-phenotype genetic evaluation, overlooking the interaction effects among component traits, which compromises breeding stability. To address this, we optimized the grouping strategy by selecting extreme E-RFI populations with consistent high-yield performance for multi-tissue transcriptomic profiling. This approach eliminates interference from production trait genetic backgrounds, precisely identifies key molecular pathways and hub genes regulating feed efficiency, and provides a robust theoretical foundation for layer breeding.

Multi-tissue integrated analysis has emerged as a pivotal strategy in livestock genetic research, offering unique advantages for deciphering genetic regulatory networks of complex traits and identifying critical biomarkers. By integrating multi-omics data (e.g., transcriptomics) across tissues and constructing cross-tissue interaction networks, this approach enables the precise identification of tissue-specific expression patterns and coordinated regulatory mechanisms, providing multi-dimensional insights into the genetic basis of complex traits. In layer breeding, this technology elucidates the key regulatory pathways influencing production performance, establishing a theoretical foundation for marker-assisted selection and precision nutritional strategies(Astuti et al., 2025; Yan et al., 2024; Liu et al., 2023).

Genetically, RFI exhibits negative correlations (−0.01 to −0.47) with its component traits(Yuan et al., 2015). Our study revealed that E-RFI showed strong phenotypic and genetic correlations with DFI_70–100__w_ (daily feed intake at 70-100 weeks), confirming its efficacy in characterizing individual feed intake requirements. Concurrently, E-RFI displayed low phenotypic and genetic correlations with EN_70-100__w_ (egg number at 70-100 weeks) and AEW_70-100__w_ (average egg weight at 70-100 weeks), demonstrating that E-RFI-based selection optimized feed efficiency without compromising core production parameters. Notably, E-RFI exhibited a strong negative genetic correlation with DWBG_70-100__w_ (daily body weight gain at 70-100 weeks). These findings propose a novel precision breeding strategy for aging layers: E-RFI-driven selection reduces feed consumption while maintaining stable egg production, achieving the breeding goal of “reducing consumption without compromising yield.”

Further studies have revealed genetic correlations between E-RFI and lipid-related traits. Improved feed efficiency (reduced E-RFI) promoted egg weight gain and fatty acid enrichment in the yolk, while decreasing yolk weight and dry matter content, suggesting that high-efficiency individuals may maintain nutrient supply by reducing absolute yolk mass while enhancing lipid concentration. These findings align with previous observations that high-RFI individuals exhibit genetic tendencies for slightly higher egg production, greater yolk weight, and greater albumen height(Wolc et al., 2013). Additionally, heavier hens produced larger eggs, with egg weight gain potentially linked to body weight increase(Summers and Leeson, 1983), consistent with the observed body weight gain and EW100 changes. Notably, dietary fat supplementation strategies synergistically reduced feed intake and enhanced egg weight, thereby lowering the feed conversion ratio (**FCR**)(Han et al., 2023).

Second, we found that the improvement of feed efficiency could enhance the synthesis and transportation efficiency of liver lipoproteins. Within a physiological range, this improvement reduced liver weight, triglyceride, and cholesterol content while increasing dry matter and free fatty acid levels. Studies have demonstrated that optimized hepatic lipid metabolism and utilization efficiency are closely linked to reduced liver weight and cholesterol levels(Oliveira et al., 2015). Consequently, elevated feed efficiency not only mitigates hepatic steatosis risk, but also supports lipid supply for follicular development, thereby promoting metabolic homeostasis and systemic balance.

Furthermore, this study elucidated the genetic link between feed efficiency and abdominal fat metabolism. The results demonstrated that improved feed efficiency increased abdominal fat weight, dry matter content, and lipid content in abdominal fat, aligning with the lipid variation patterns observed in egg yolks. This indicates that high-efficiency individuals enhance their lipid storage capacity in adipose tissue to sustain energy reserves, reflecting a nutrient redistribution mechanism through systemic coordination. These findings parallel Marks et al.’s report of increased abdominal fat deposition and water intake in high-efficiency hens(Marks and Pesti, 1984). Additionally, improved feed efficiency elevated abdominal fat, dry matter and lipid content(Li et al., 2024).

Multi-tissue joint analysis revealed metabolic pathways and module genes that are closely associated with feed efficiency in the liver, duodenum, and cecum. Notably, although pancreatic tissue showed the most pronounced DEGs and GO/KEGG pathway enrichment, its weighted gene co-expression network analysis did not yield any modules significantly correlated with E-RFI. Further KEGG enrichment analysis (Fig. 2) revealed that pancreatic DEGs were predominantly enriched in highly conserved, cross-tissue pathways such as muscle contraction, cell adhesion, and neuroregulation. They collectively maintain pancreatic structural stability and participate in stress responses(Li et al., 2025; Woo et al., 2020). Only the MAPK and calcium signaling pathways were linked to the pancreas-specific secretory functions, these two pathways maintain pancreatic cell proliferation, insulin secretion, and inflammatory responses through dynamic equilibrium. Once imbalanced, they directly contribute to the pathogenesis of diabetes, pancreatitis, and other related disorders(Ahn et al., 2015; Li et al., 2021; Martinez et al., 2022). This dichotomy-where functional pathways exhibit broad tissue distribution alongside tissue-specific specialization-likely weakened the biological relevance between pancreatic co-expression modules and E-RFI. Consequently, this phenomenon of functional compensation through other digestive organs (such as the intestine) may explain why these modules are not identified as significant modules in WGCNA screening.

Subsequently, we found that although there is no direct intersection among the module genes, their protein-protein interaction networks exhibit significant cross-tissue functional synergy, constructing a multi-dimensional regulatory network through specific molecular mechanisms to jointly regulate feed efficiency. The 22 hub genes identified through screening, including *SHMT1, OAT, CPT1A,* and *CYCS*, exhibited cross-tissue enrichment characteristics in key processes, such as substance and energy metabolism and protein homeostasis, forming a multidimensional regulatory architecture. Functional analysis of key genes revealed that *SOCS3* maintains the dynamic balance of cytokine and hormone signaling pathways through negative regulation(Ban et al., 2017); *SDHB* regulates mitochondrial energy metabolism balance, and its functional deficiency can lead to decreased metabolic efficiency(Chen et al., 2023); *ACO2* catalyzes the entry of citrate into the tricarboxylic acid cycle, regulating feed conversion efficiency through energy metabolism(Wu et al., 2022), low-RFI steers enhance feed efficiency by upregulating *ACO2* expression in ruminal epithelial cells, thereby boosting the tricarboxylic acid cycle activity and energy production(Elolimy et al., 2018); *CPT1A* mediates the rate-limiting step of long-chain fatty acid oxidation, regulating intestinal energy metabolism, lipid absorption, and intestinal barrier function(Chu et al., 2023), *CPT1A* expression is upregulated in high-efficiency individuals, showing a significant negative correlation with RFI(Jin et al., 2019); *CYCS* drives mitochondrial energy generation as an electron carrier(Dunislawska et al., 2023); *GAPDH* regulates the glycerol metabolic process(Luo et al., 2022); *PSMA1*, as a core subunit of the proteasome, is involved in the regulation of protein catabolism(Olukosi et al., 2019).

This study systematically analyzed the genetic patterns of residual feed intake and related traits at 100 weeks of age in individuals aged 70 to 100 weeks. However, a longitudinal comparative study with key indicators at 70 weeks of age has not been conducted. Subsequent research could supplement phenotypic and genotypic data across time points to enhance the dynamic association analysis. At the molecular level, although multiple candidate genes associated with residual feed intake have been identified through multi-tissue transcriptome analysis, experimental validation is still lacking and needs to be supplemented.

## Conclusion

Our analysis of E-RFI and related traits revealed that E-RFI, egg number (70-100 weeks), and average egg weight exhibited moderate to high heritability, whereas lipid metabolism indicators (triglycerides, free fatty acids, and cholesterol) showed moderate to low heritability. Furthermore, we identified 22 candidate genes for E-RFI, including *SHMT1, OAT, SOCS3, NT5C1B, CYCS,* and *SDHB*. Multi-tissue synergy analysis further demonstrated that these genes not only exhibit tissue-specific functions but also actively participate in cross-tissue biological pathways, representing a network of interconnected biochemical processes. This study precisely identifies functional candidate genes influencing RFI in late-phase laying hens, offering novel mechanistic insights into feed efficiency regulation. These findings establish a crucial theoretical foundation for breeding programs targeting enhanced feed efficiency in poultry production.

Availability of data and materials

RNA-Seq data are available from the SRA under accession number SUB15530077 (hypothalamus, pituitary, liver, pancreas, duodenum, ileum, and cecum).

These data have not yet been released, and the reviewer links are as follows:

Transcriptome: https://dataview.ncbi.nlm.nih.gov/object/PRJNA1305156?reviewer=fjm2b67kg3gmeu6cn7d4avf5t9

## CRediT authorship contribution statement

**Yuejie Han:** Formal analysis, Writing – original draft. **Fangren Lan:** Formal analysis. **Ronglang Cai:** Software. **Wenxin Zhang:** Resources. **Daqing Dai:** Formal analysis. **Xinwei Jiang:** Methodology. **Junnan Zhang:** Resources. **Ning Yang:** Funding acquisition, Resources. **Congjiao Sun:** Data curation, Funding acquisition, Methodology, Project administration.

## Disclosures

The authors declare that they have no competing interests.

## Appendix Supplementary materials

Image, application 1

Image, application 2

## References

[bib0001] Ahn C., An B.-S., Jeung E.-B. (2015). Streptozotocin induces endoplasmic reticulum stress and apoptosis via disruption of calcium homeostasis in mouse pancreas. Mol. Cellular Endocrinol..

[bib0002] Ashburner M., Ball C.A., Blake J.A., Botstein D., Butler H., Cherry J.M., Davis A.P., Dolinski K., Dwight S.S., Eppig J.T., Harris M.A., Hill D.P., Issel-Tarver L., Kasarskis A., Lewis S., Matese J.C., Richardson J.E., Ringwald M., Rubin G.M., Sherlock G. (2000). Gene ontology: tool for the unification of biology. Nat Genet..

[bib0003] Astuti P.K., Sárkány P., Wanjala G., Bagi Z., Kusza S. (2025). A systematic review on the trend of transcriptomic study in livestock: an effort to unwind the complexity of adaptation in a climate change environment. Heliyon.

[bib0004] Bain M.M., Nys Y., Dunn I.C. (2016). Increasing persistency in lay and stabilising egg quality in longer laying cycles. What are the challenges?. Br. Poult. Sci..

[bib0005] Ban Q., Hui W., Cheng F., Liu D., Liu X. (2017). Effect of chicken leptin recptor short hairpin RNA on expression of JAK2, STAT3, SOCS3 and CPT1 genes in chicken preadipocytes. Anim. Sci. J..

[bib0006] Chen D., Shen F., Liu J., Tang H., Zhang K., Teng X., Yang F. (2023). The protective effect of luteolin on chicken spleen lymphocytes from ammonia poisoning through mitochondria and balancing energy metabolism disorders. Poult. Sci..

[bib0007] Chen Y., Gondro C., Quinn K., Herd R.M., Parnell P.F., Vanselow B. (2011). Global gene expression profiling reveals genes expressed differentially in cattle with high and low residual feed intake. Anim. Genet..

[bib0008] Chu Y., Zheng Y., Li Y., Gui S., Zhao J., Zhao Y., Chen X. (2023). Dietary supplementation of magnolol alleviates fatty liver hemorrhage syndrome in postpeak xinhua laying hens via regulation of liver lipid metabolism. Poultry Sci..

[bib0009] Dunislawska A., Bełdowska A., Yatsenko O., Siwek M. (2023). Effect of prebiotics administered during embryo development on mitochondria in intestinal and immune tissues of adult broiler chickens. Poultry Sci..

[bib0010] Elolimy A.A., Abdelmegeid M.K., McCann J.C., Shike D.W., Loor J.J. (2018). Residual feed intake in beef cattle and its association with carcass traits, ruminal solid-fraction bacteria, and epithelium gene expression. J. Anim. Sci. Biotechnol..

[bib0011] Han G.P., Kim J.H., Lee J.H., Kim H.W., Kil D.Y. (2023). Research note: effect of increasing fat supplementation in diets on productive performance, egg quality, and fatty liver incidence in laying hens throughout the entire laying cycle. Poultry Sci..

[bib0012] Huang D.W., Sherman B.T., Lempicki R.A. (2009). Systematic and integrative analysis of large gene lists using DAVID bioinformatics resources. Nat. Protoc..

[bib0013] Huang Q., Wen C., Yan W., Sun C., Gu S., Zheng J., Yang N. (2022). Comparative analysis of the characteristics of digestive organs in broiler chickens with different feed efficiencies. Poult. Sci..

[bib0014] Jin S., Yang L., Fan X., Wu M., Xu Y., Chen X., Lin Z., Geng Z. (2019). Effect of divergence in residual feed intake on expression of lipid metabolism-related genes in the liver of meat-type ducks1. J. Anim. Sci..

[bib0015] Kanehisa M. (2000). KEGG: kyoto encyclopedia of genes and genomes. Nucleic Acids Res..

[bib0016] Karimi P., Bakhtiarizadeh M.R., Salehi A., Izadnia H.R. (2022). Transcriptome analysis reveals the potential roles of long non-coding RNAs in feed efficiency of chicken. Sci. Rep..

[bib0017] Kechin A., Boyarskikh U., Kel A., Filipenko M. (2017). cutPrimers: a new tool for accurate cutting of primers from reads of targeted next generation sequencing. J. Comput. Biol..

[bib0018] Kennedy B.W., van der Werf J.H., Meuwissen T.H. (1993). Genetic and statistical properties of residual feed intake. J. Anim. Sci..

[bib0019] Kim D., Langmead B., Salzberg S.L. (2015). HISAT: a fast spliced aligner with low memory requirements. Nat. Methods.

[bib0020] Kim D., Paggi J.M., Park C., Bennett C., Salzberg S.L. (2019). Graph-based genome alignment and genotyping with HISAT2 and HISAT-genotype. Nat. Biotechnol..

[bib0021] Koch R., Gregory K., Chambers D., Swiger L. (1963). Efficiency of feed use in beef cattle. J. Anim. Sci..

[bib0022] Kovaka S., Zimin A.V., Pertea G.M., Razaghi R., Salzberg S.L., Pertea M. (2019). Transcriptome assembly from long-read RNA-seq alignments with StringTie2. Genome Biol..

[bib0023] Langfelder P., Horvath S. (2008). WGCNA: an R package for weighted correlation network analysis. BMC Bioinf..

[bib0024] Langfelder P., Horvath S. (2008). WGCNA: an R package for weighted correlation network analysis. BMC Bioinf..

[bib0025] Li C., Cui L., Zhang L., Yang L., Zhuo Y., Cui J., Cui N., Zhang S. (2021). Saikosaponin D attenuates pancreatic injury through suppressing the apoptosis of acinar cell via modulation of the MAPK signaling pathway. Front. Pharmacol..

[bib0026] Li H., Zhang Z., Shi Z., Zhou S., Nie S., Yu Y., Zhang L., Sun Y., Fang C., Hu J., Niu Y., Schuck K., Wang L., Jiang K., Lu Z., Kahlert C., Roth S., Loos M., Herr I., Sunami Y., Kleeff J., Friess H., Reichert M., Dantes Z., Zou X., Michalski C.W., Shen S., Kong B. (2025). Disrupting AGR2/IGF1 paracrine and reciprocal signaling for pancreatic cancer therapy. Cell Rep. Med..

[bib0027] Li Q., Xu G., Yang D., Tu Y., Zhang J., Ma T., Diao Q. (2024). Effects of feed ingredients with different protein-to-fat ratios on growth, slaughter performance and fat deposition of small-tail han lambs. Animals.

[bib0028] Liu L., Zhang G., Qu G., Liu B., Zhang X., Li G., Jin N., Li C., Bai J., Zhao C. (2023). Effects of dietary lactobacillus rhamnosus GG supplementation on the production performance, egg quality, eggshell ultrastructure, and lipid metabolism of late-phase laying hens. BMC Veterinary Res..

[bib0030] Luo N., Shu J., Yuan X., Jin Y., Cui H., Zhao G., Wen J. (2022). Differential regulation of intramuscular fat and abdominal fat deposition in chickens. BMC Genom..

[bib0031] Marks H.L., Pesti G.M. (1984). The roles of protein level and diet form in water consumption and abdominal fat pad deposition of broilers. Poult. Sci..

[bib0032] Martinez C., Maschio D.A., De Fontes C.C., Vanzela E.C., Benfato I.D., Gazarini M.L., Carneiro E.M., De Oliveira C.A.M., Collares-Buzato C.B., Carvalho C.P.F. (2022). Early decrease in Cx36 is associated with increased cell adhesion molecules (CAMs) junctional content in mouse pancreatic islets after short-term high-fat diet feeding. Ann. Anat. - Anat. Anz..

[bib0033] Montanholi Y., Fontoura A., Swanson K., Coomber B., Yamashiro S., Miller S. (2013). Small intestine histomorphometry of beef cattle with divergent feed efficiency. Acta Vet. Scand..

[bib0034] Oliveira P.R.B.de, Costa C.A.da, Bem G.F.de, Cordeiro V.S.C., Santos I.B., Carvalho L.C.R.M.de, Conceição E.P.S.da, Lisboa P.C., Ognibene D.T., Sousa P.J.C., Martins G.R., Silva A.J.R.da, Moura R.S.de, Resende A.C. (2015). Euterpe oleracea mart.-derived polyphenols protect mice from diet-induced obesity and fatty liver by regulating hepatic lipogenesis and cholesterol excretion. PLoS One.

[bib0035] Olukosi O.A., Kuijk S.J.A.van, Han Y. (2019). Sulfate and hydroxychloride trace minerals in poultry diets – comparative effects on egg production and quality in laying hens, and growth performance and oxidative stress response in broilers. Poultry Sci..

[bib0036] Pertea M., Kim D., Pertea G.M., Leek J.T., Salzberg S.L. (2016). Transcript-level expression analysis of RNA-seq experiments with HISAT, StringTie and ballgown. Nat. Protoc..

[bib0037] Pertea M., Pertea G.M., Antonescu C.M., Chang T.-C., Mendell J.T., Salzberg S.L. (2015). StringTie enables improved reconstruction of a transcriptome from RNA-seq reads. Nat. Biotechnol..

[bib0038] Reyer H., Shirali M., Ponsuksili S., Murani E., Varley P.F., Jensen J., Wimmers K. (2017). Exploring the genetics of feed efficiency and feeding behaviour traits in a pig line highly selected for performance characteristics. Mol. Genet. Genomics.

[bib0039] Robinson M.D., McCarthy D.J., Smyth G.K. (2010). edgeR : a bioconductor package for differential expression analysis of digital gene expression data. Bioinformatics.

[bib0040] Saintilan R., Mérour I., Brossard L., Tribout T., Dourmad J.Y., Sellier P., Bidanel J., Van Milgen J., Gilbert H. (2013). Genetics of residual feed intake in growing pigs: relationships with production traits, and nitrogen and phosphorus excretion traits1. J. Anim. Sci..

[bib0041] Sharma V.K., Kundu S.S., Datt C., Prusty S., Kumar M., Sontakke U.B. (2018). Buffalo heifers selected for lower residual feed intake have lower feed intake, better dietary nitrogen utilisation and reduced enteric methane production. J. Anim. Physiol. Anim. Nutr. (Berl.).

[bib0042] Sherman B.T., Hao M., Qiu J., Jiao X., Baseler M.W., Lane H.C., Imamichi T., Chang W. (2022). DAVID: a web server for functional enrichment analysis and functional annotation of gene lists (2021 update). Nucleic Acids Res..

[bib0043] Song J., Huang M., Shi X., Li X., Chen X., He Z., Li J., Xu G., Zheng J. (2021). T329S mutation in the FMO3 gene alleviates lipid metabolic diseases in chickens in the late laying period. Anim. : Open Access J. MDPI.

[bib0044] Summers J., Leeson S. (1983). Factors influencing early egg size. Poult. Sci..

[bib0045] Szklarczyk D., Kirsch R., Koutrouli M., Nastou K., Mehryary F., Hachilif R., Gable A.L., Fang T., Doncheva N.T., Pyysalo S., Bork P., Jensen L.J., von Mering C. (2023). The STRING database in 2023: protein–protein association networks and functional enrichment analyses for any sequenced genome of interest. Nucleic Acids Res..

[bib0046] Tang J., Kong D., Cui Q., Wang K., Zhang D., Gong Y., Wu G. (2018). Prognostic genes of breast cancer identified by gene Co-expression network analysis. Front. Oncol..

[bib0047] Thompson O., Von Meyenn F., Hewitt Z., Alexander J., Wood A., Weightman R., Gregory S., Krueger F., Andrews S., Barbaric I., Gokhale P.J., Moore H.D., Reik W., Milo M., Nik-Zainal S., Yusa K., Andrews P.W. (2020). Low rates of mutation in clinical grade human pluripotent stem cells under different culture conditions. Nat. Commun..

[bib0048] Urgessa O.E., Woldesemayat A.A. (2023). OMICs approaches and technologies for understanding low-high feed efficiency traits in chicken: implication to breeding. Anim. Biotechnol..

[bib0049] Van Eerden E., Van Den Brand H., Parmentier H.K., De Jong M.C.M., Kemp B. (2004). Phenotypic selection for residual feed intake and its effect on humoral immune responses in growing layer hens. Poult. Sci..

[bib0050] Van Kaam J., Groenen M., Bovenhuis H., Veenendaal A., Vereijken A., Van Arendonk J. (1999). Whole genome scan in chickens for quantitative trait loci affecting growth and feed efficiency. Poultry Sci..

[bib0051] Wolc A., Arango J., Jankowski T., Settar P., Fulton J.E., O’Sullivan N.P., Fernando R., Garrick D.J., Dekkers J.C.M. (2013). Pedigree and genomic analyses of feed consumption and residual feed intake in laying hens. Poult. Sci..

[bib0052] Woo J., Sudhir P., Zhang Q. (2020). Pancreatic tissue proteomics unveils key proteins, pathways, and networks associated with type 1 diabetes. Proteom. – Clin. Appl..

[bib0053] Wu P., Zhou K., Zhang J., Ling X., Zhang X., Li P., Zhang L., Wei Q., Zhang T., Xie K., Zhang G. (2022). Transcriptome integration analysis at different embryonic ages reveals key lncRNAs and mRNAs for chicken skeletal muscle. Front. Veterinary Sci..

[bib0054] Xiao C., Deng J., Zeng L., Sun T., Yang Z., Yang X. (2021). Transcriptome analysis identifies candidate genes and signaling pathways associated with feed efficiency in xiayan chicken. Front. Genet..

[bib0055] Xu Z., Ji C., Zhang Y., Zhang Z., Nie Q., Xu J., Zhang D., Zhang X. (2016). Combination analysis of genome-wide association and transcriptome sequencing of residual feed intake in quality chickens. BMC Genom..

[bib0056] Yan W., Sun C., Wen C., Ji C., Zhang D., Yang N. (2019). Relationships between feeding behaviors and performance traits in slow-growing yellow broilers. Poult. Sci..

[bib0057] Yan Y., Zhu S., Jia M., Chen X., Qi W., Gu F., Valencak T.G., Liu J.-X., Sun H.-Z. (2024). Advances in single-cell transcriptomics in animal research. J. Anim. Sci. Biotechnol..

[bib0058] Yang J., Lee S.H., Goddard M.E., Visscher P.M. (2011). GCTA: a tool for genome-wide complex trait analysis. Am. J. Hum. Genet..

[bib0059] Yang L., He T., Xiong F., Chen X., Fan X., Jin S., Geng Z. (2020). Identification of key genes and pathways associated with feed efficiency of native chickens based on transcriptome data via bioinformatics analysis. BMC Genomics.

[bib0060] Yang L.B. (2020). Procedia Comput. Sci., Proceedings of the 3rd International Conference on Mechatronics and Intelligent Robotics (ICMIR-2019).

[bib0061] Yi G., Yuan J., Bi H., Yan W., Yang N., Qu L. (2015). In-depth duodenal transcriptome survey in chickens with divergent feed efficiency using RNA-seq. PLoS One.

[bib0062] Yuan J., Dou T., Ma M., Yi G., Chen S., Lujiang Q.u., Shen M., Liang Q.u., Wang K., Yang N. (2015). Genetic parameters of feed efficiency traits in laying period of chickens. Poult. Sci..

[bib0063] Yuan J., Zhao J., Sun Y., Wang Y., Li Y., Ni A., Zong Y., Ma H., Wang P., Shi L., Chen J. (2023). The mRNA-lncRNA landscape of multiple tissues uncovers key regulators and molecular pathways that underlie heterosis for feed intake and efficiency in laying chickens. Genet. Sel. Evol..

[bib0064] Zhou Q., Lan F., Gu S., Li G., Wu G., Yan Y., Li X., Jin J., Wen C., Sun C., Yang N. (2023). Genetic and microbiome analysis of feed efficiency in laying hens. Poult. Sci..

