# Supplementary material for: Integrated multi-tissue transcriptomics reveals cross-tissue regulatory networks and hub genes regulating feed efficiency in aging chicken
Source: Poult Sci. 2025 Aug 21;104(11):105711. doi: 10.1016/j.psj.2025.105711 (PMC12410010; doi:10.1016/j.psj.2025.105711)
Supplement: Supplementary file 1 [file mmc1.pdf]

Figure S1. PCA plot and volcano plot of 7 tissues in Group 1-3.

Hypothalamus

(A)

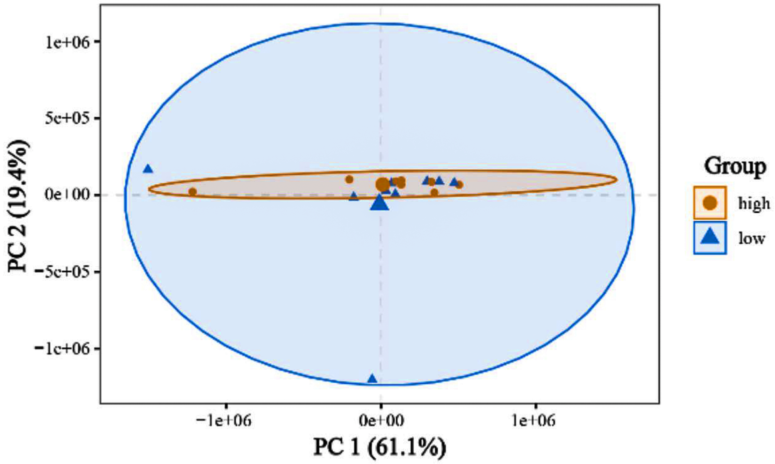

(B)

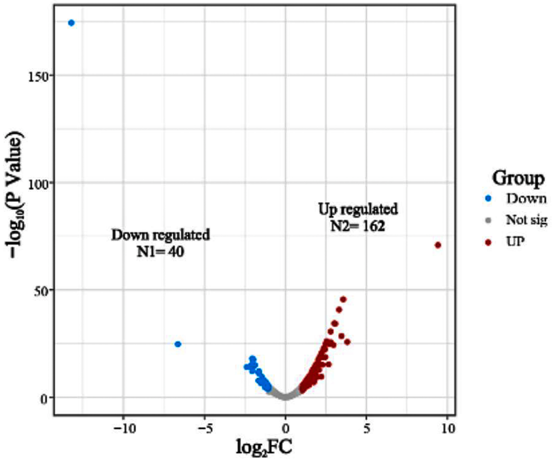

Liver

(A)

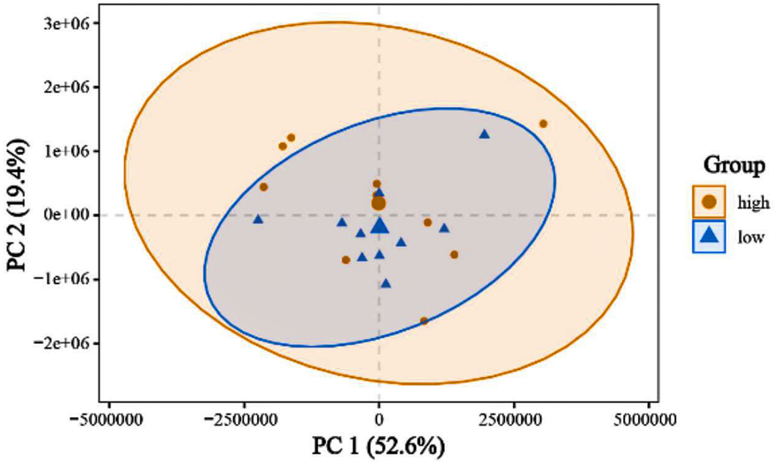

(B)

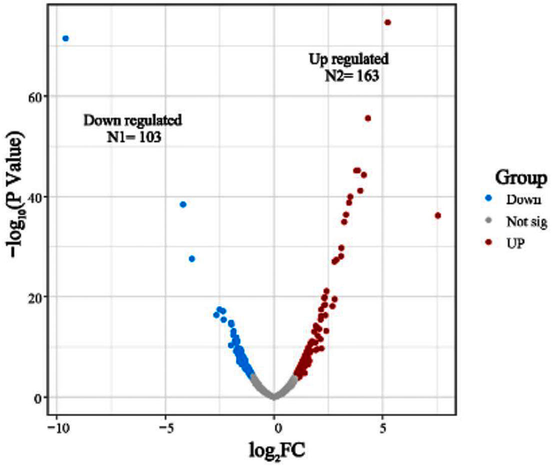

Pituitary

(A) (B)

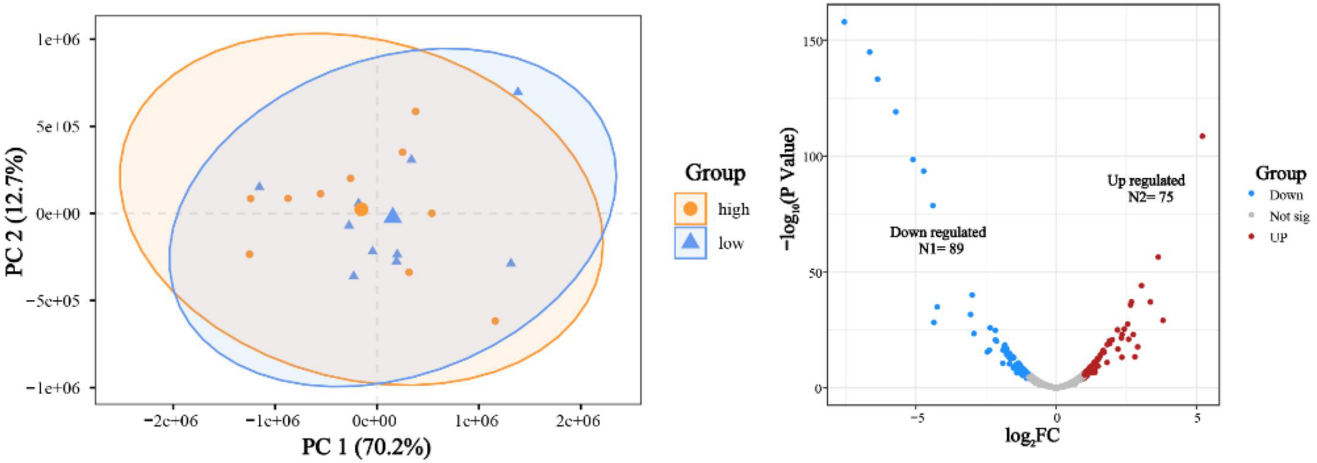

Pancreas

(A) (B)

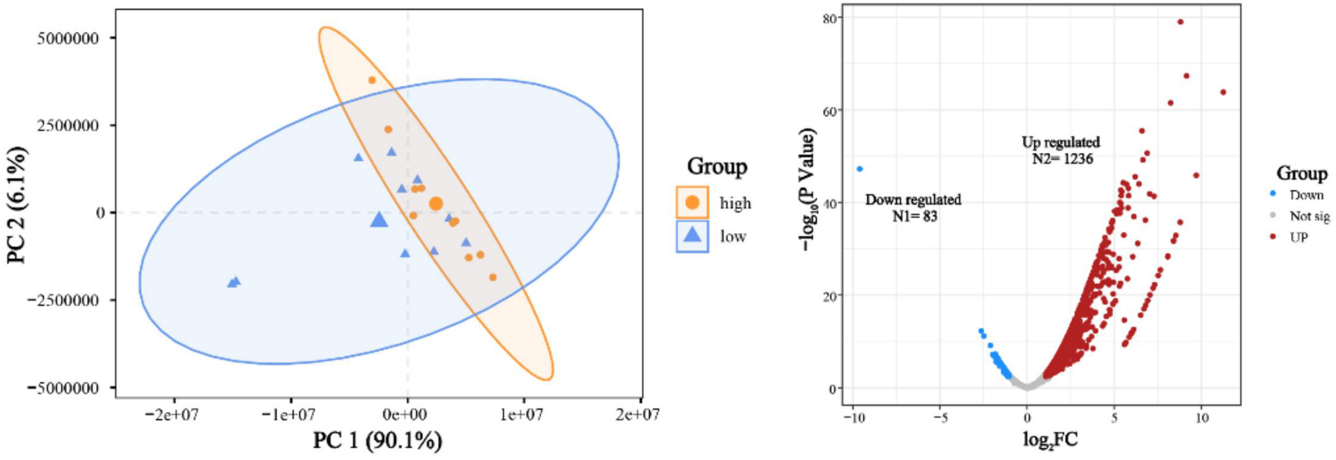

Duodenum

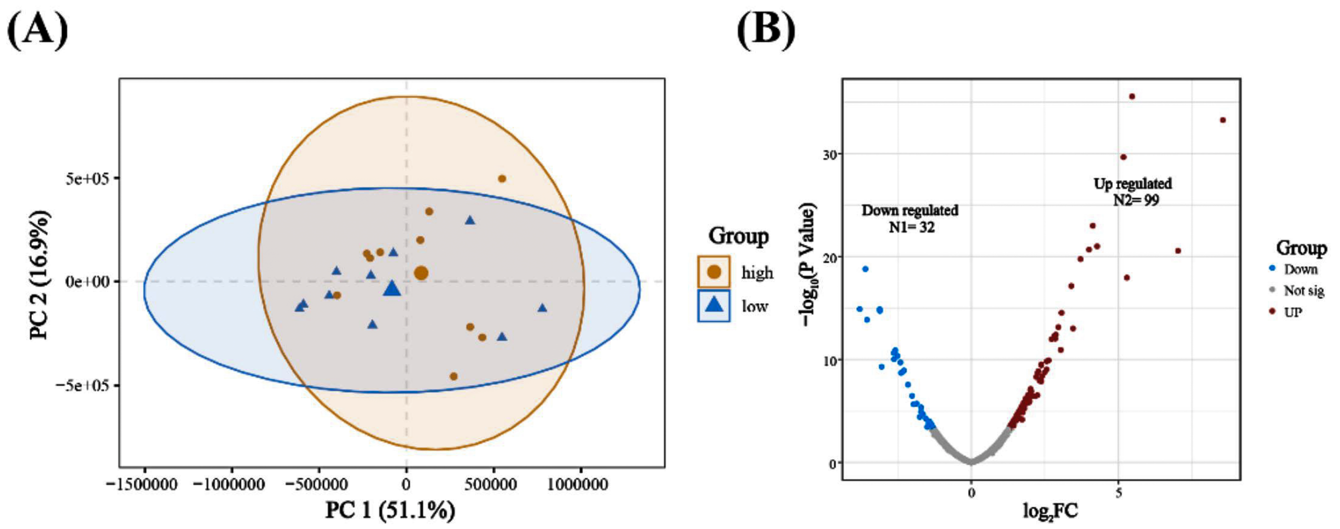

Cecum

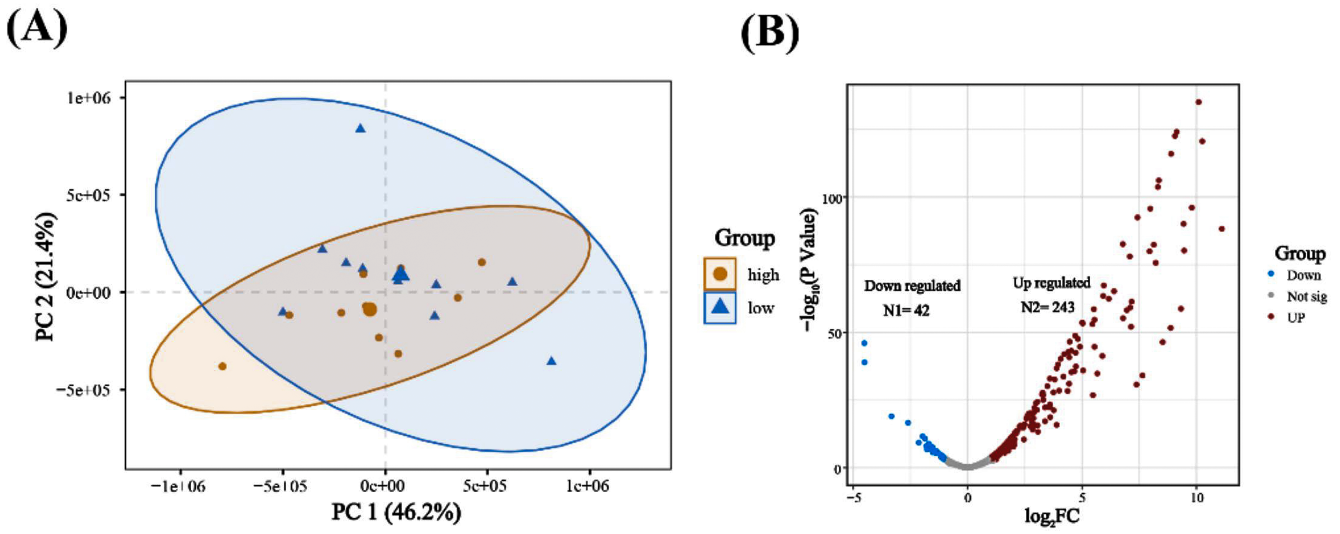

Ileum

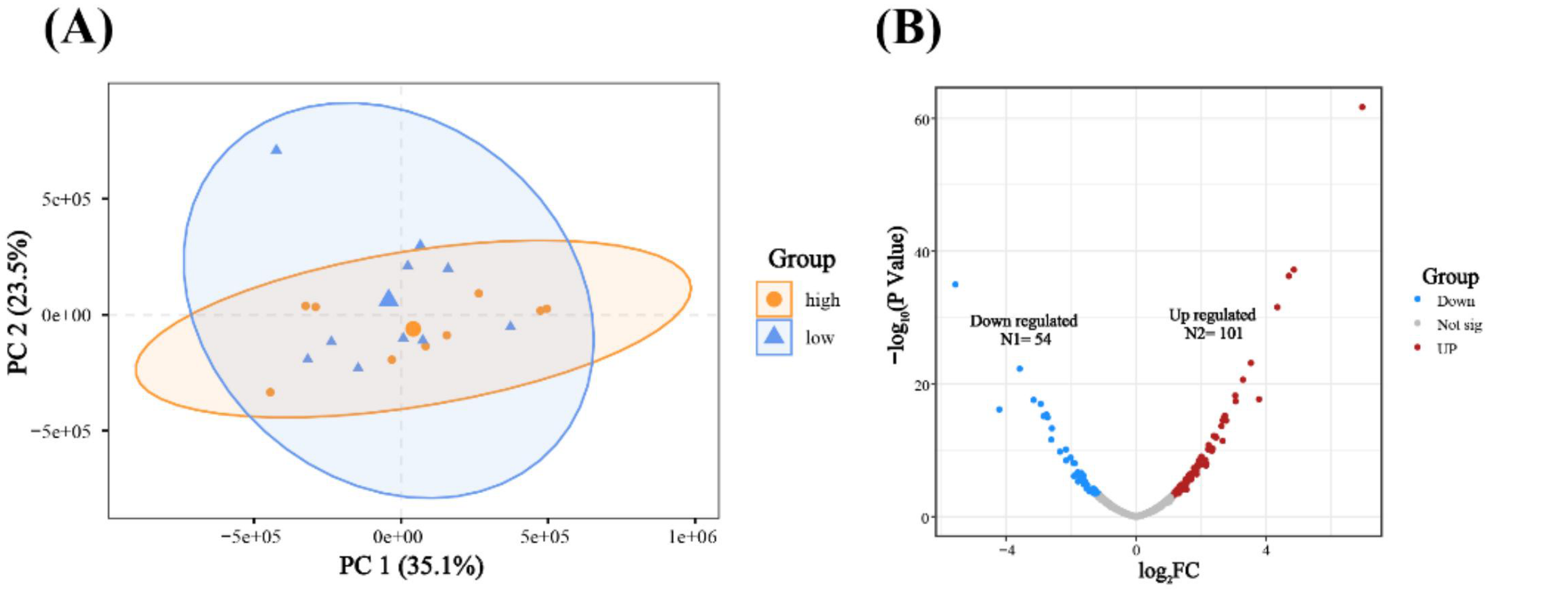

Note: (A) PCA diagram of each tissue in groups 1-3. Orange and blue dots represent individuals in Group 3 and Group 1 respectively. (B) Volcano plot of differentially expressed genes in each tissue of groups 1-3. Blue and red dots represent down-regulated genes and up-regulated genes respectively. The X and Y axes represent $\log_2$FC and -$\log_{10}$(P Value) respectively. One individual in group 3 was missing from the hypothalamic sequencing results.

Figure S2. GO entry diagram and KEGG pathway diagram of 7 tissues in groups 1-3.

Hypothalamus

(A)

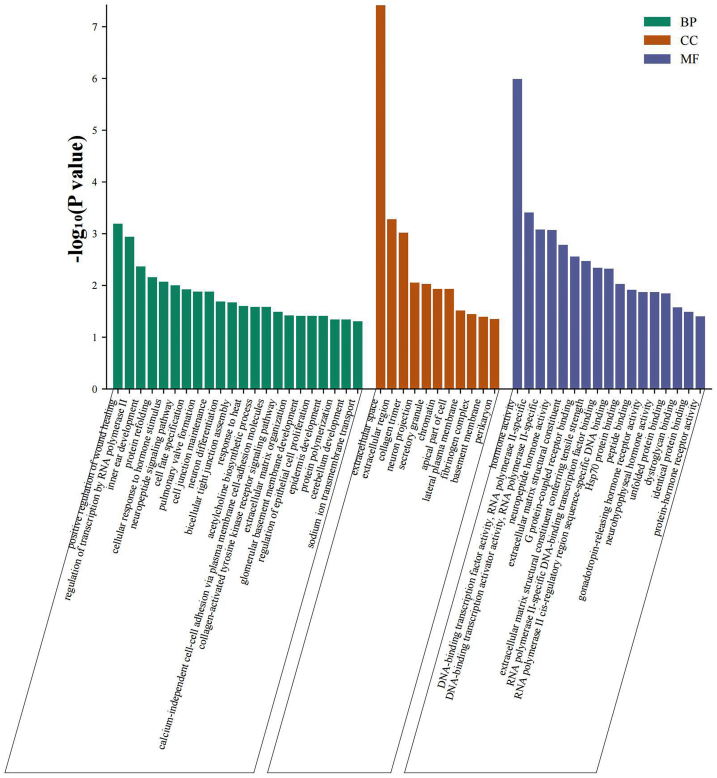

Pituitary

(A)

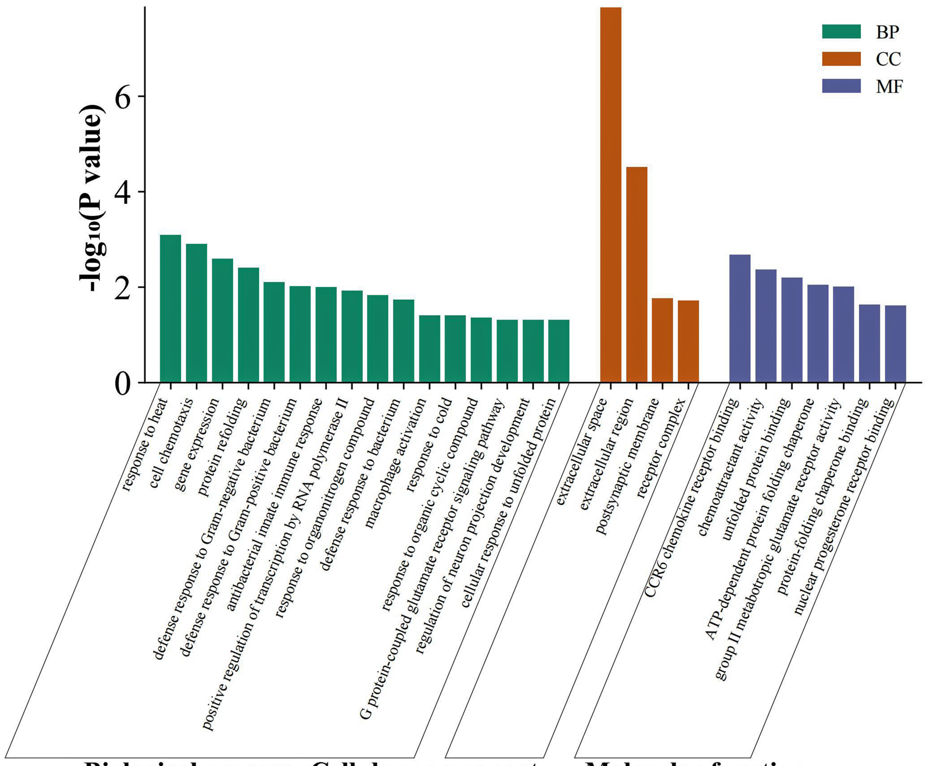

(B)

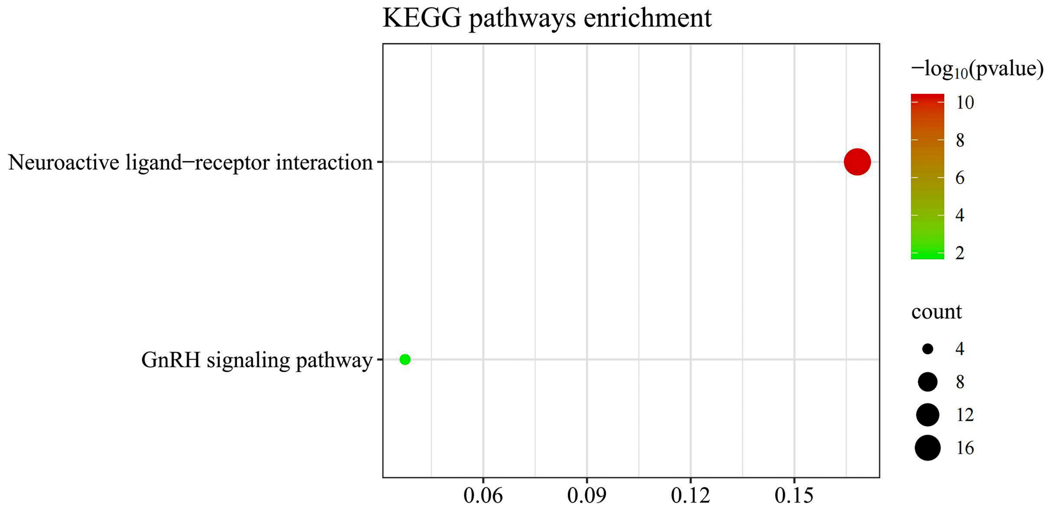

KEGG pathways enrichment
Neuroactive ligand−receptor interaction
GnRH signaling pathway
0.06
0.09
0.12
0.15
−log10(pvalue)
10
8
6
4
2
count
4
8
12
16

(B)

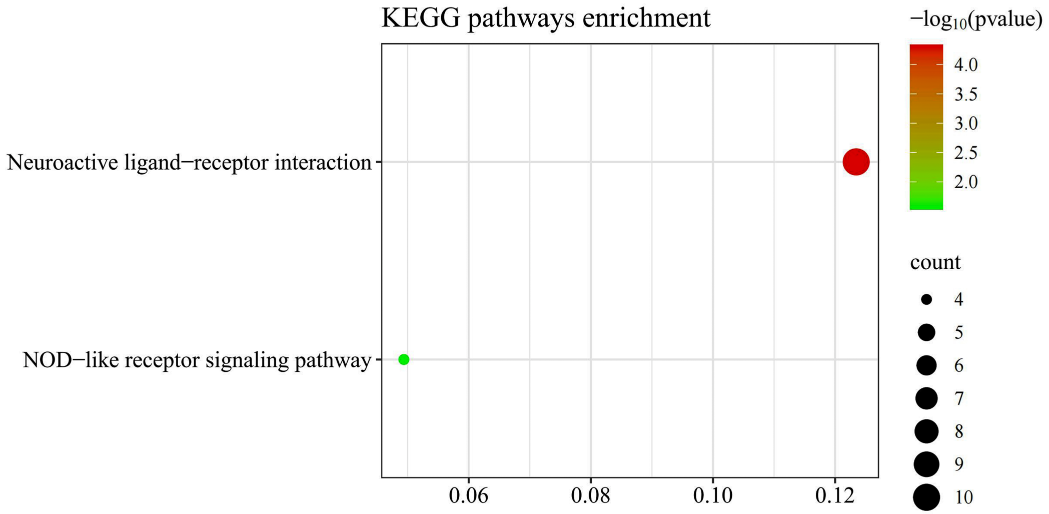

KEGG pathways enrichment
Neuroactive ligand−receptor interaction
NOD−like receptor signaling pathway
0.06
0.08
0.10
0.12
−log10(pvalue)
4.0
3.5
3.0
2.5
2.0
count
4
5
6
7
8
9
10

Liver

(A)

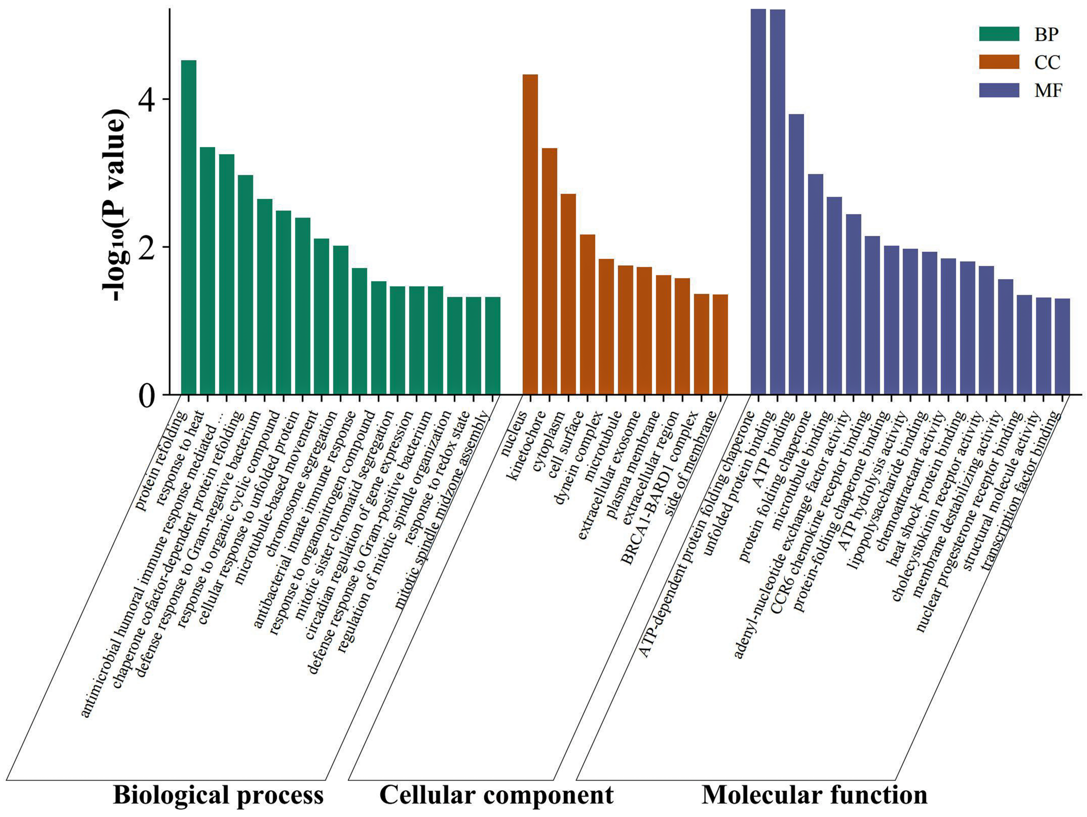

-log10(P value)
0
2
4
BP
CC
MF
protein refolding
response to heat
antimicrobial humoral immune response mediated ...
chaperone cofactor-dependent protein refolding
defense response to Gram-negative bacterium
response to organic cyclic compound
cellular response to unfolded protein
microtubule-based movement
chromosome segregation
antibacterial innate immune response
response to organonitrogen compound
mitotic sister chromatid segregation
circadian regulation of gene expression
defense response to Gram-positive bacterium
regulation of mitotic spindle organization
response to redox state
mitotic spindle midzone assembly
nucleus
kinetochore
cytoplasm
cell surface
dynein complex
microtubule
extracellular exosome
plasma membrane
extracellular region
BRCA1-BARD1 complex
side of membrane
ATP-dependent protein folding chaperone
unfolded protein binding
ATP binding
protein folding chaperone
microtubule binding
adenyl-nucleotide exchange factor activity
CCR6 chemokine receptor binding
protein-folding chaperone binding
ATP hydrolysis activity
lipopolysaccharide binding
chemoattractant activity
heat shock protein binding
cholecystokinin receptor activity
membrane destabilizing activity
nuclear progesterone receptor binding
structural molecule activity
transcription factor binding
Biological process
Cellular component
Molecular function

(B)

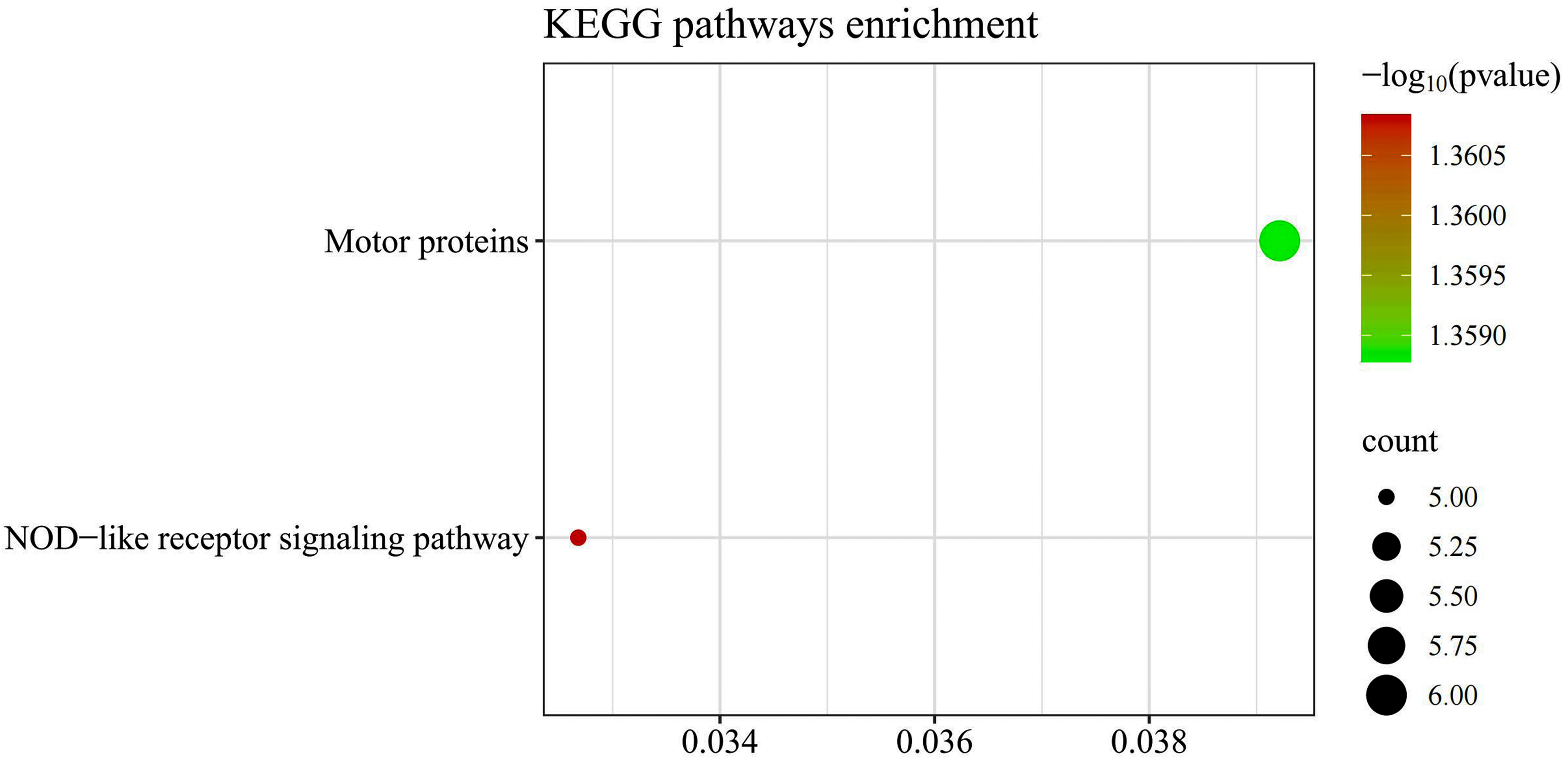
KEGG pathways enrichment
Motor proteins
NOD−like receptor signaling pathway
0.034
0.036
0.038
−log10(pvalue)
1.3605
1.3600
1.3595
1.3590
count
5.00
5.25
5.50
5.75
6.00

Pancreas

(A)

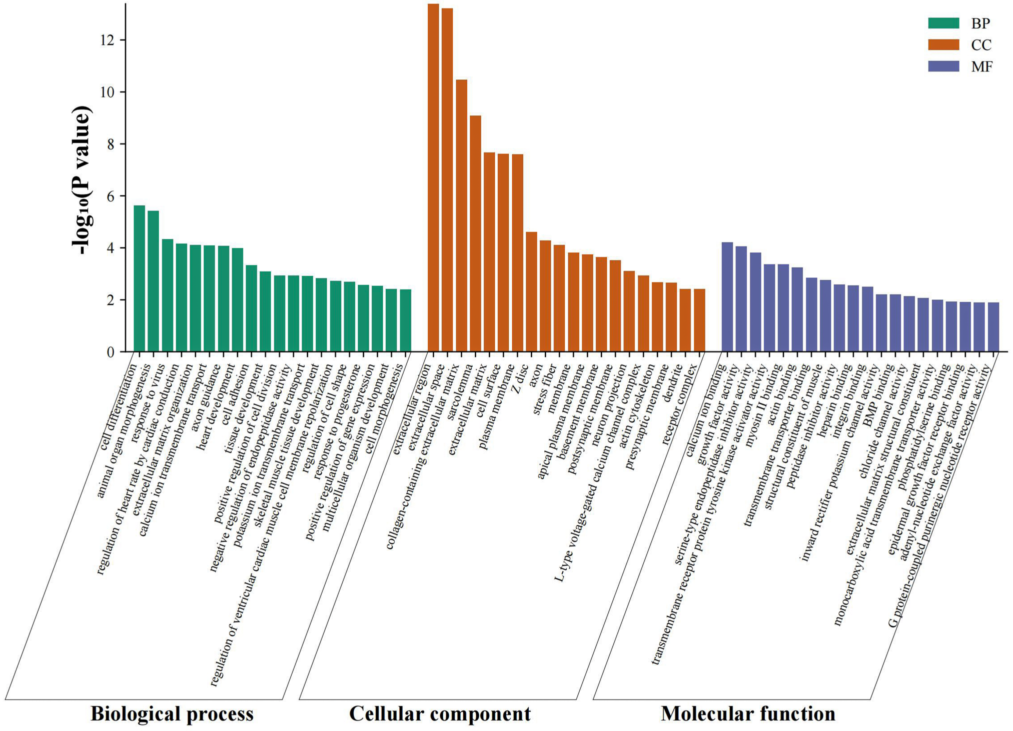

Duodenum

(A)

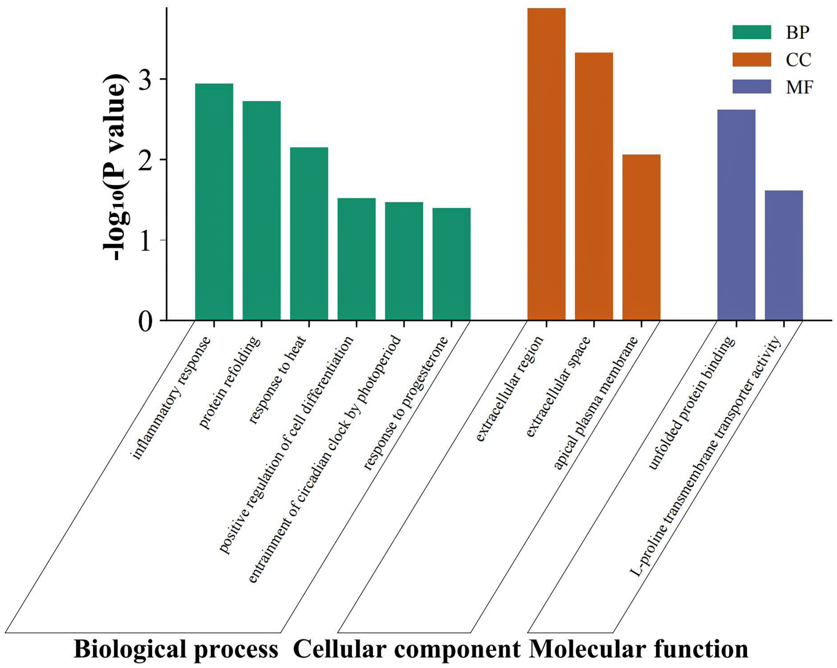

(B)

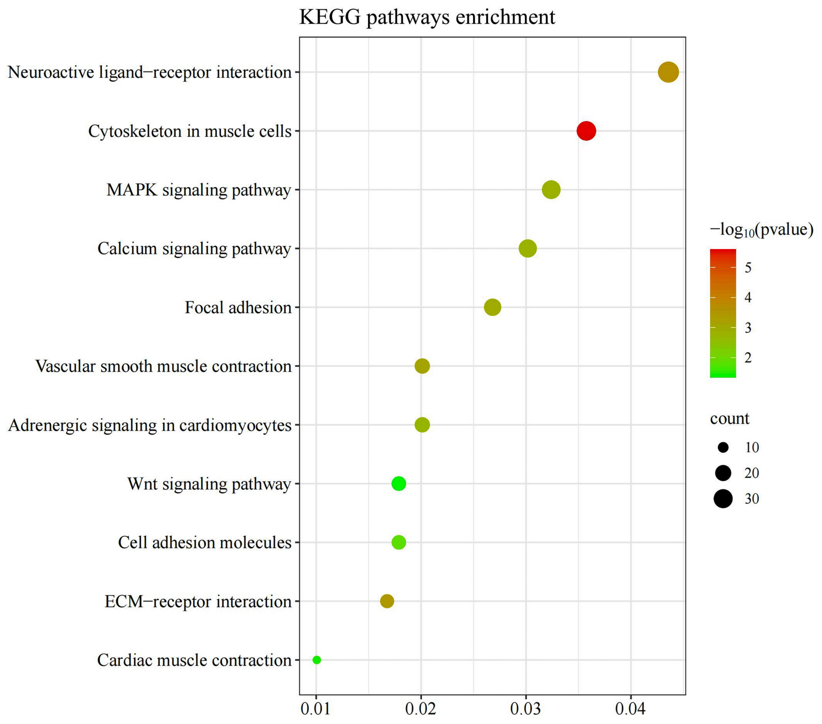
KEGG pathways enrichment
Neuroactive ligand−receptor interaction
Cytoskeleton in muscle cells
MAPK signaling pathway
Calcium signaling pathway
Focal adhesion
Vascular smooth muscle contraction
Adrenergic signaling in cardiomyocytes
Wnt signaling pathway
Cell adhesion molecules
ECM−receptor interaction
Cardiac muscle contraction
0.01
0.02
0.03
0.04
−log10(pvalue)
5
4
3
2
count
10
20
30

(B)

(B)

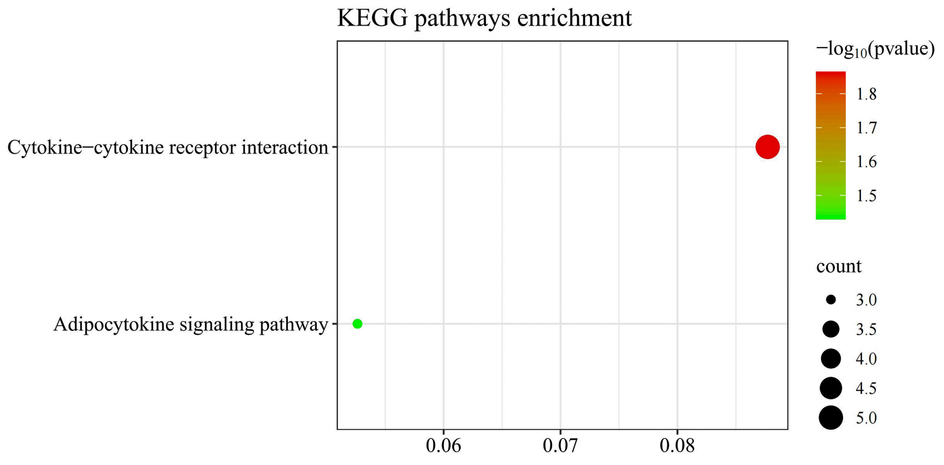
KEGG pathways enrichment
Cytokine−cytokine receptor interaction
Adipocytokine signaling pathway
0.06
0.07
0.08
−log10(pvalue)
1.8
1.7
1.6
1.5
count
3.0
3.5
4.0
4.5
5.0

Ileum

(A)

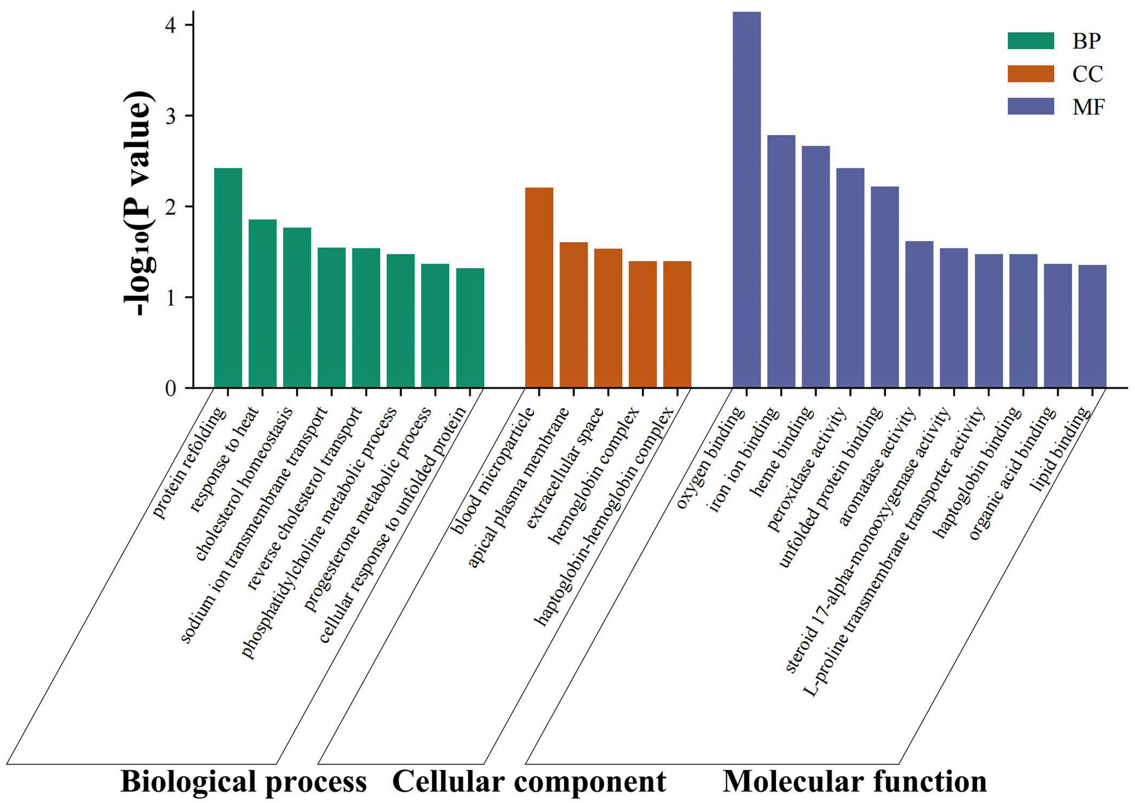

-log10(P value)
0
1
2
3
4
BP
CC
MF
protein refolding
response to heat
cholesterol homeostasis
sodium ion transmembrane transport
reverse cholesterol transport
phosphatidylcholine metabolic process
progesterone metabolic process
cellular response to unfolded protein
blood microparticle
apical plasma membrane
extracellular space
hemoglobin complex
haptoglobin-hemoglobin complex
oxygen binding
iron ion binding
heme binding
peroxidase activity
unfolded protein binding
aromatase activity
steroid 17-alpha-monooxygenase activity
L-proline transmembrane transporter activity
haptoglobin binding
organic acid binding
lipid binding
Biological process
Cellular component
Molecular function

Cecum

(A)

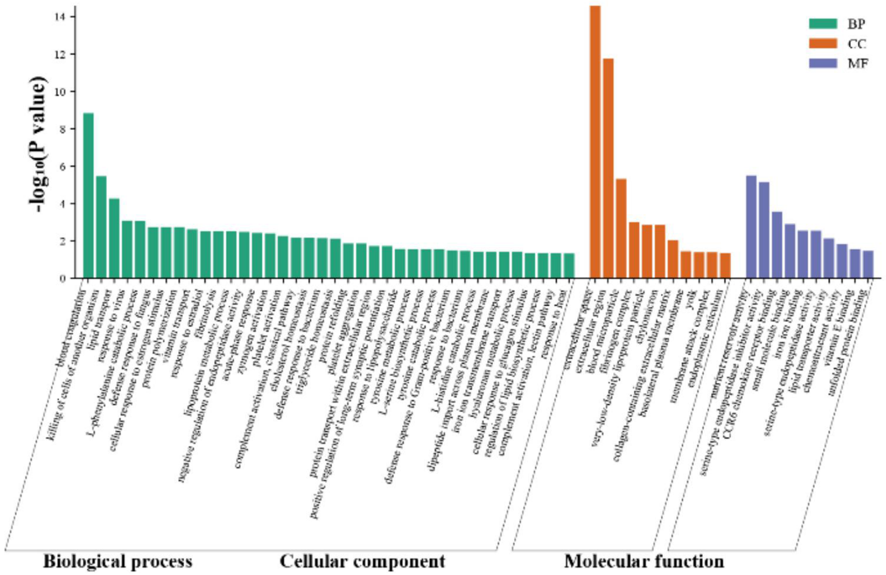

(B)

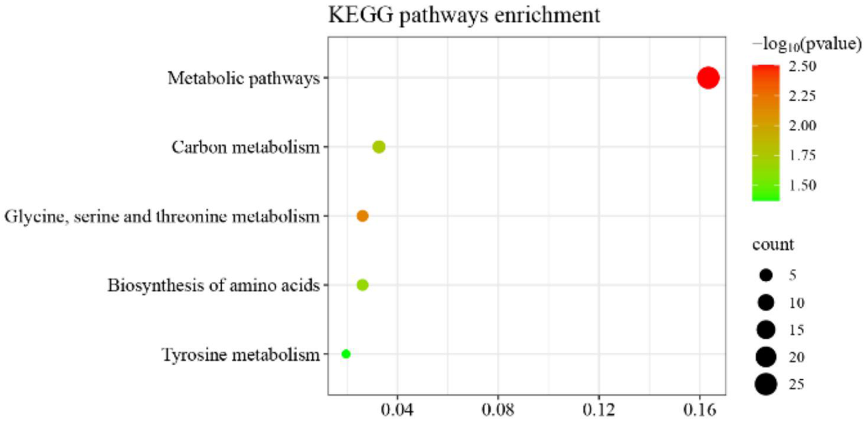

Note: (A) Map of significantly enriched GO entries in each tissue (P<0.05). The green, orange and blue bars represent BP, CC and MF; respectively. Among them, BP, CC and MF of pancreatic tissue each display the Top 20 entry. (B)Diagram of significantly enriched KEGG pathways in each tissue (P<0.05). Among them, the KEGG pathway was not significantly enriched in the lleum.

Figure S3. The result graphs of WGCNA analysis for each tissue.

Hypothalamus

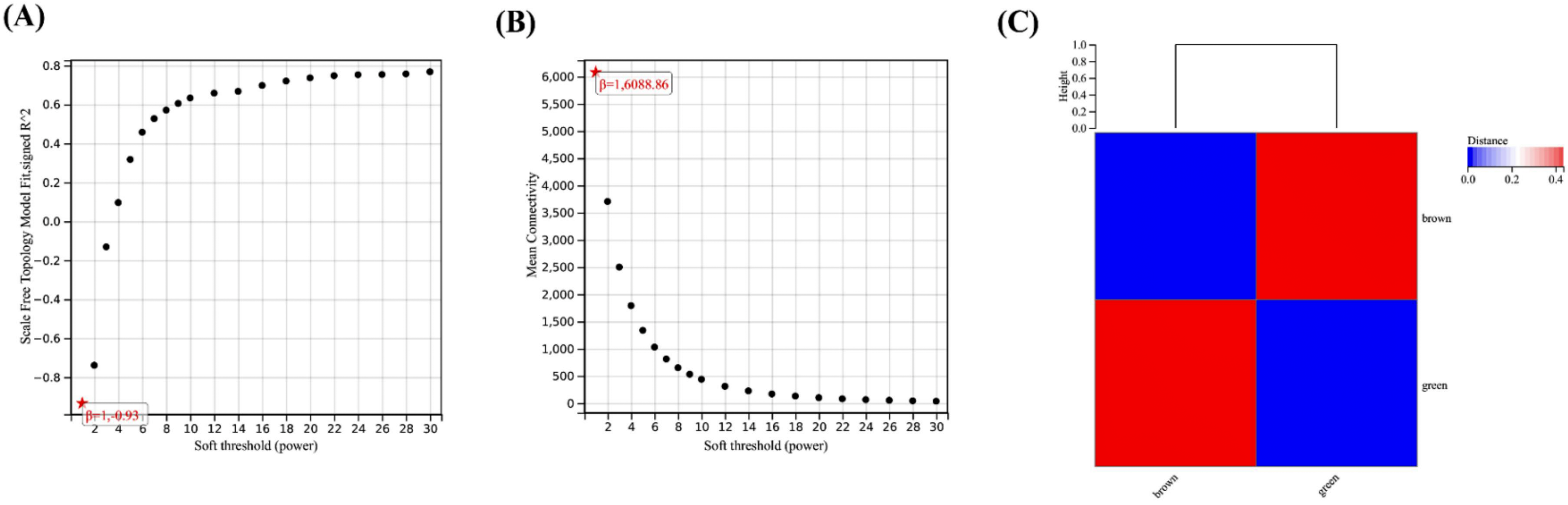

(D)

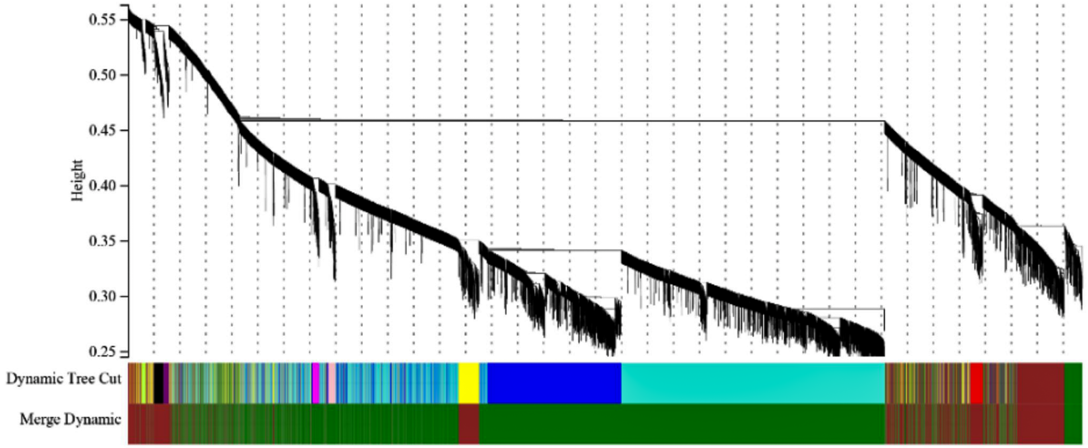

Height
0.55
0.50
0.45
0.40
0.35
0.30
0.25
Dynamic Tree Cut
Merge Dynamic

(E)

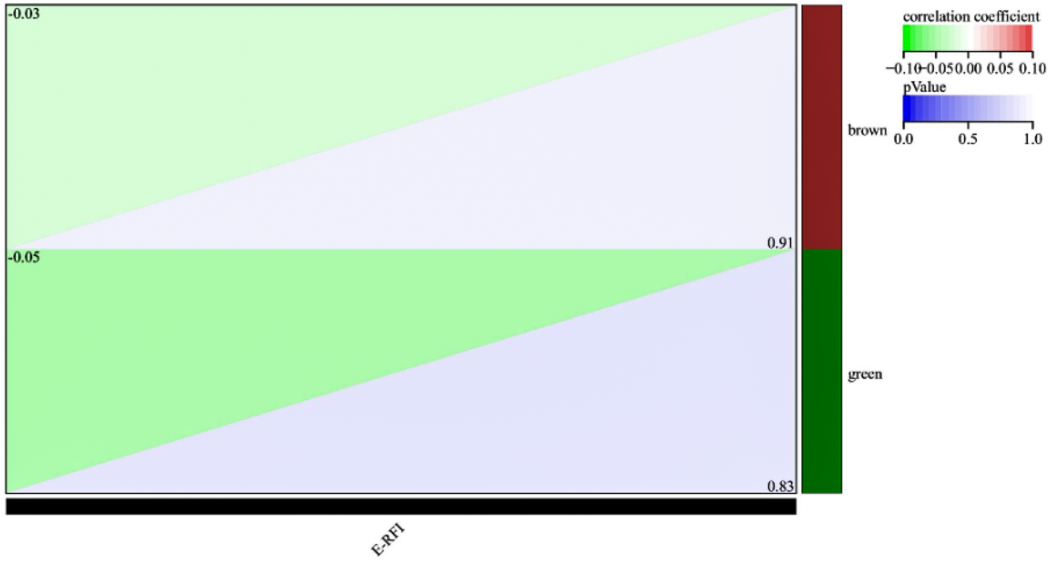

-0.03
0.91
-0.05
0.83
brown
green
E-RFI
correlation coefficient
−0.10 −0.05 0.00 0.05 0.10
pValue
0.0 0.5 1.0

Pituitary

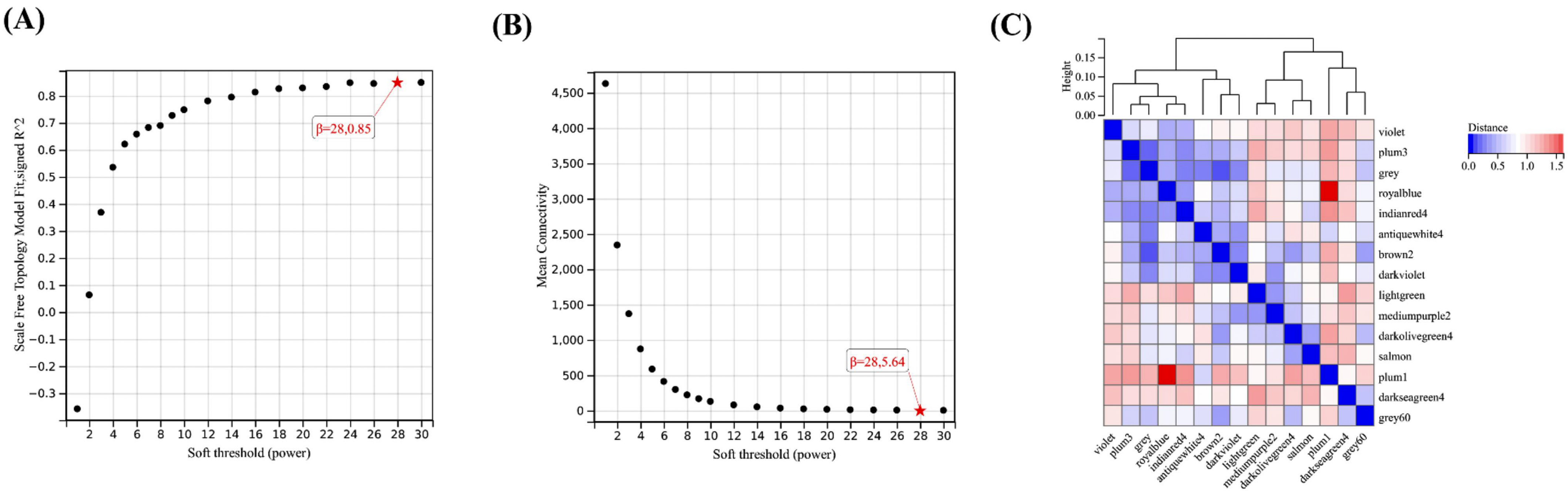
(A)
Scale Free Topology Model Fit,signed R^2
Soft threshold (power)
β=28,0.85
(B)
Mean Connectivity
Soft threshold (power)
β=28,5.64
(C)
Height
Distance
violet
plum3
grey
royalblue
indianred4
antiquewhite4
brown2
darkviolet
lightgreen
mediumpurple2
darkolivegreen4
salmon
plum1
darkseagreen4
grey60

(D)

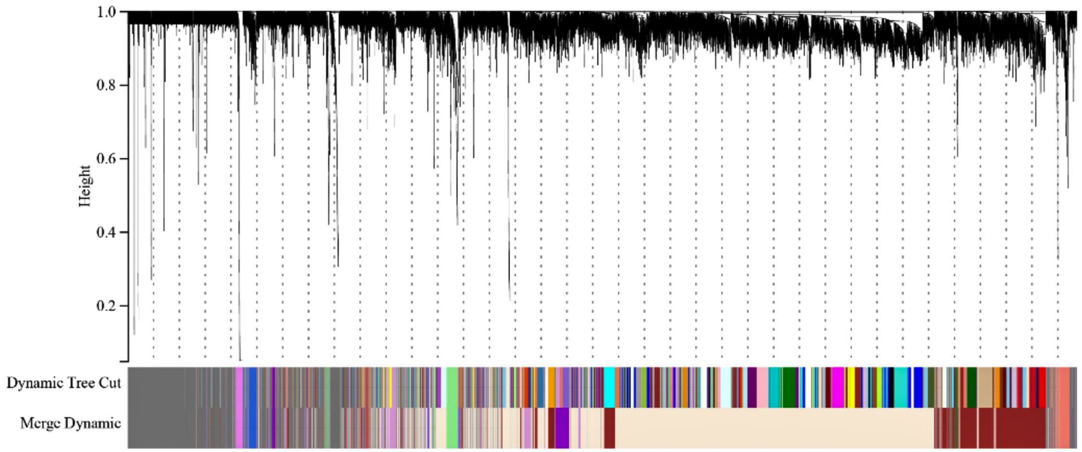
1.0
0.8
0.6
0.4
0.2
Height
Dynamic Tree Cut
Merge Dynamic

(E)

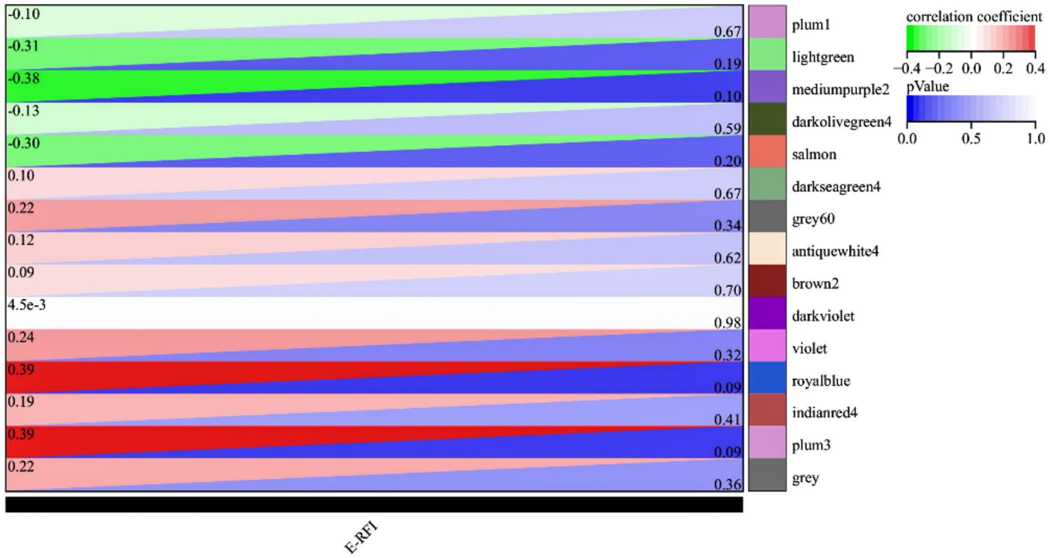
-0.10
0.67
-0.31
0.19
-0.38
0.10
-0.13
0.59
-0.30
0.20
0.10
0.67
0.22
0.34
0.12
0.62
0.09
0.70
4.5e-3
0.98
0.24
0.32
0.39
0.09
0.19
0.41
0.39
0.09
0.22
0.36
plum1
lightgreen
mediumpurple2
darkolivegreen4
salmon
darkseagreen4
grey60
antiquewhite4
brown2
darkviolet
violet
royalblue
indianred4
plum3
grey
correlation coefficient
−0.4 −0.2 0.0 0.2 0.4
pValue
0.0 0.5 1.0
E-RFI

Liver

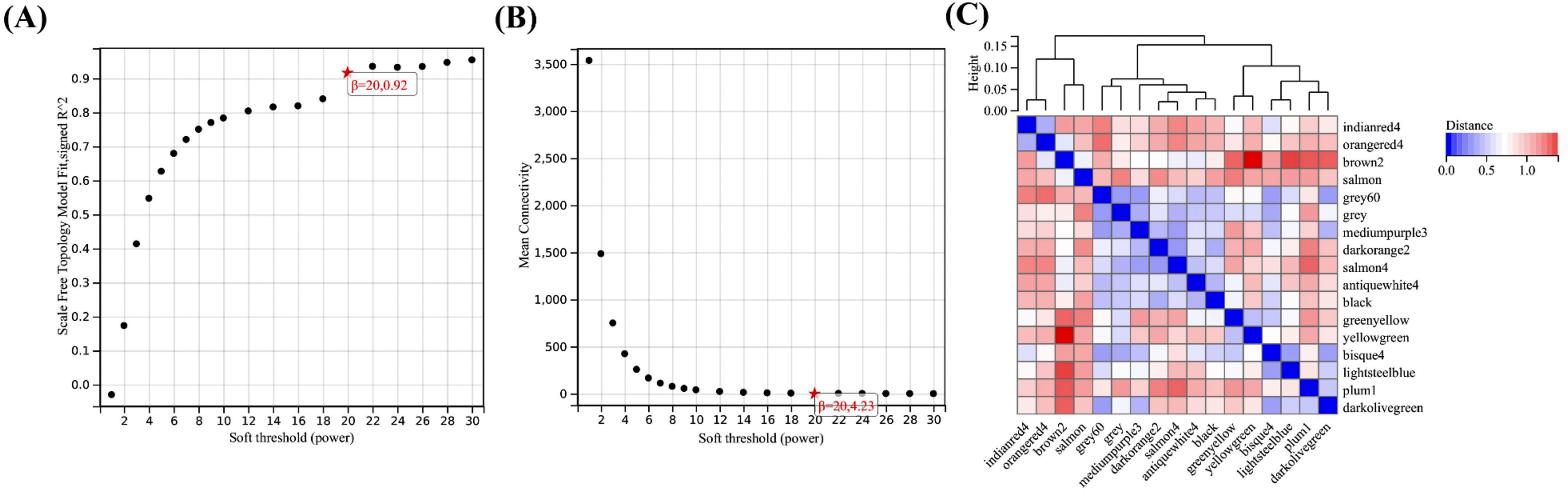
(A)
Scale Free Topology Model Fit,signed R^2
Soft threshold (power)
β=20,0.92
(B)
Mean Connectivity
Soft threshold (power)
β=20,4.23
(C)
Height
Distance
indianred4
orangered4
brown2
salmon
grey60
grey
mediumpurple3
darkorange2
salmon4
antiquewhite4
black
greenyellow
yellowgreen
bisque4
lightsteelblue
plum1
darkolivegreen

(D)

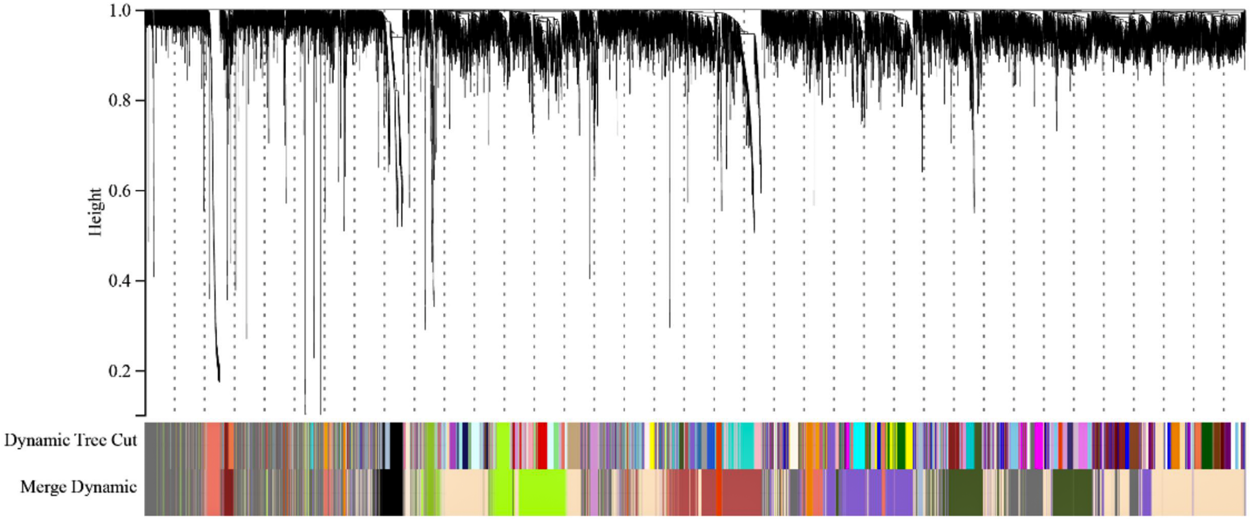

1.0
0.8
0.6
0.4
0.2
Height
Dynamic Tree Cut
Merge Dynamic

(E)

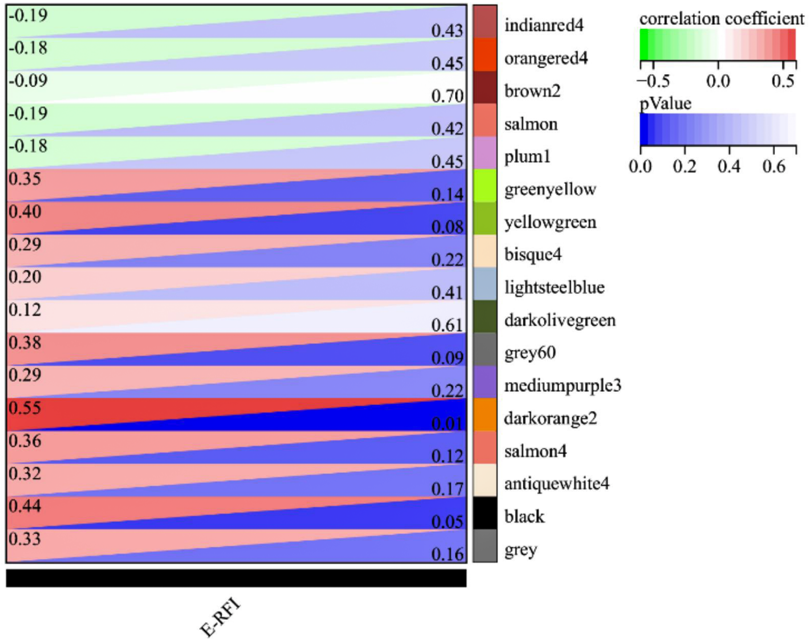

-0.19 0.43
-0.18 0.45
-0.09 0.70
-0.19 0.42
-0.18 0.45
0.35 0.14
0.40 0.08
0.29 0.22
0.20 0.41
0.12 0.61
0.38 0.09
0.29 0.22
0.55 0.01
0.36 0.12
0.32 0.17
0.44 0.05
0.33 0.16
indianred4
orangered4
brown2
salmon
plum1
greenyellow
yellowgreen
bisque4
lightsteelblue
darkolivegreen
grey60
mediumpurple3
darkorange2
salmon4
antiquewhite4
black
grey
correlation coefficient
−0.5 0.0 0.5
pValue
0.0 0.2 0.4 0.6
E-RFI

Pancreas

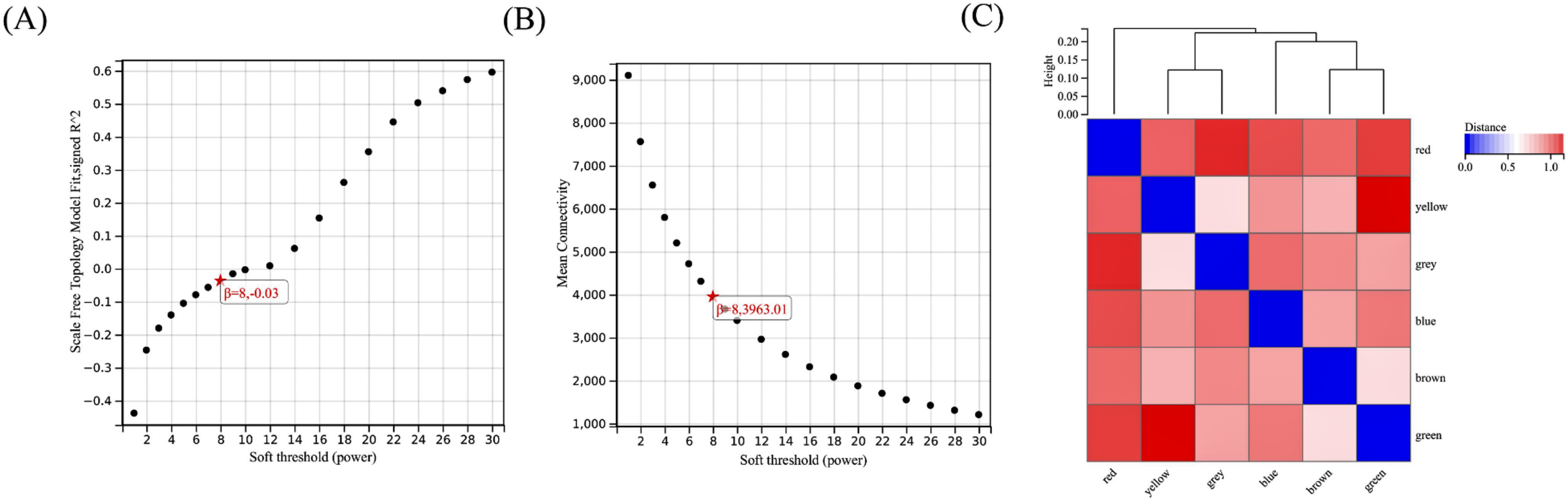

(A)
Scale Free Topology Model Fit,signed R^2
β=8,-0.03
Soft threshold (power)
(B)
Mean Connectivity
β=8,3963.01
Soft threshold (power)
(C)
Height
Distance
red
yellow
grey
blue
brown
green

(D)

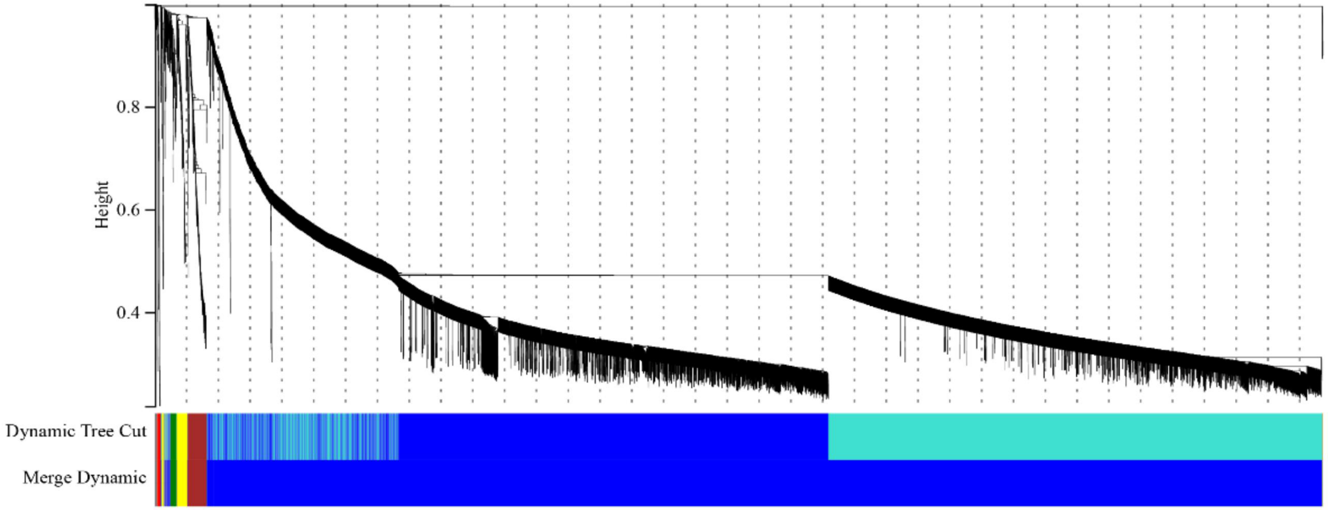
Height
0.8
0.6
0.4
Dynamic Tree Cut
Merge Dynamic

(E)

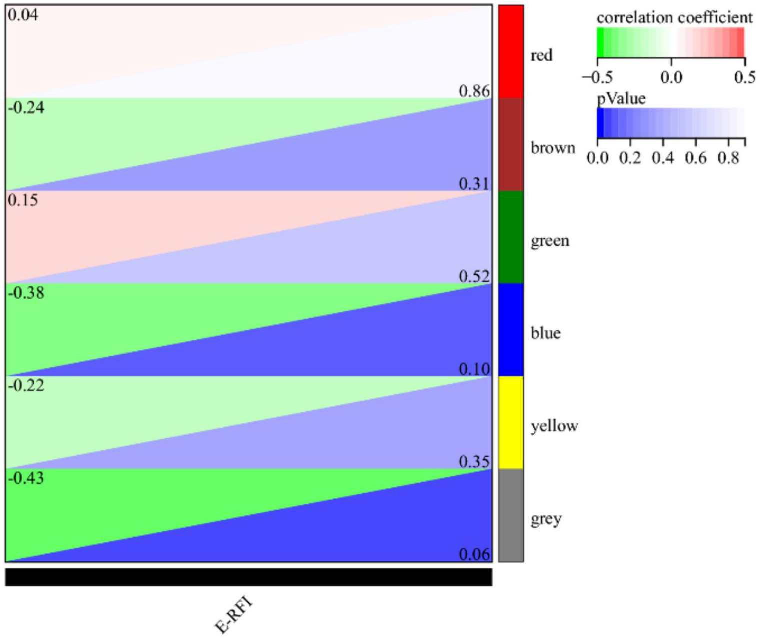
0.04
0.86
-0.24
0.31
0.15
0.52
-0.38
0.10
-0.22
0.35
-0.43
0.06
red
brown
green
blue
yellow
grey
correlation coefficient
−0.5 0.0 0.5
pValue
0.0 0.2 0.4 0.6 0.8
E-RFI

Duodenum

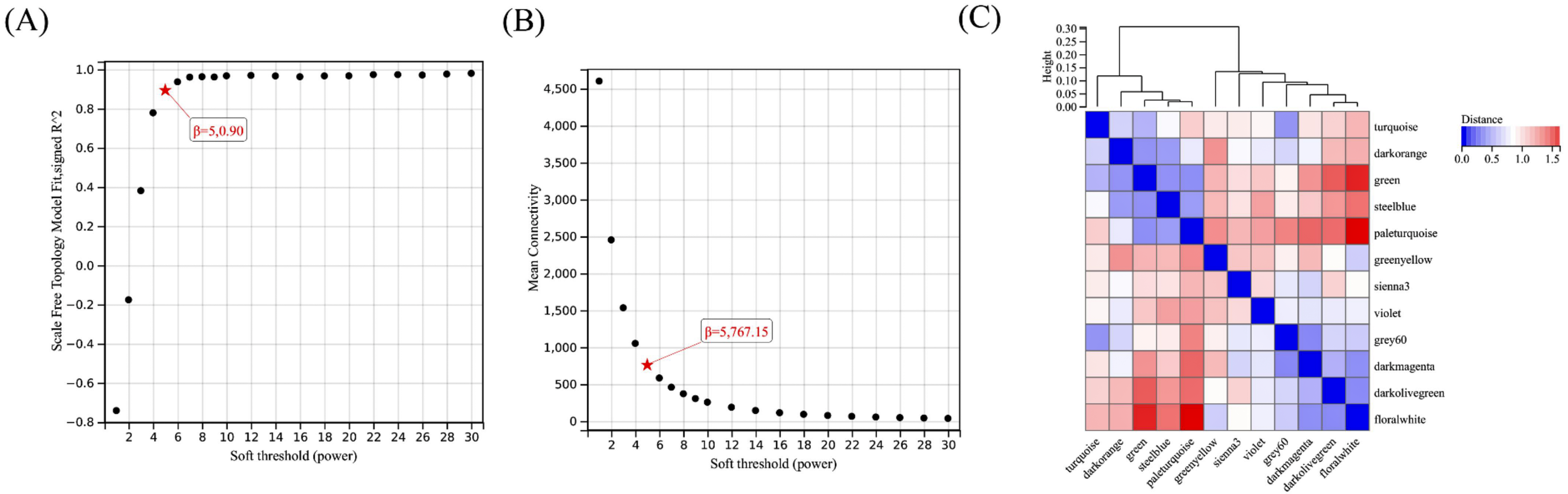

(A)
Scale Free Topology Model Fit,signed R^2
β=5,0.90
Soft threshold (power)
(B)
Mean Connectivity
β=5,767.15
Soft threshold (power)
(C)
Height
Distance
turquoise
darkorange
green
steelblue
paleturquoise
greenyellow
sienna3
violet
grey60
darkmagenta
darkolivegreen
floralwhite

(D)

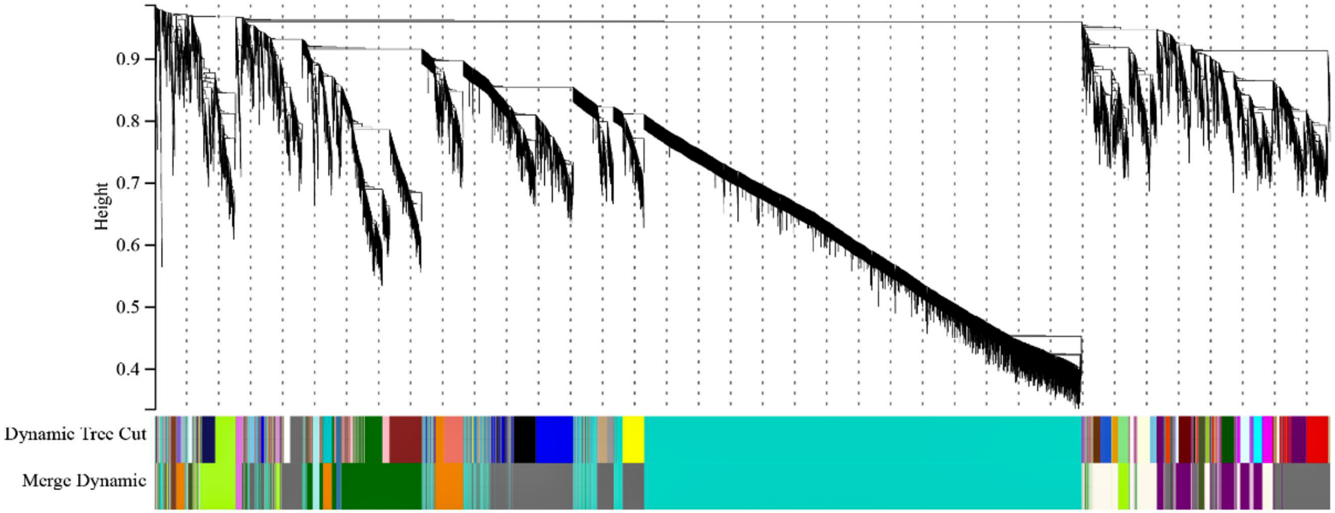
Height
0.9
0.8
0.7
0.6
0.5
0.4
Dynamic Tree Cut
Merge Dynamic

(E)

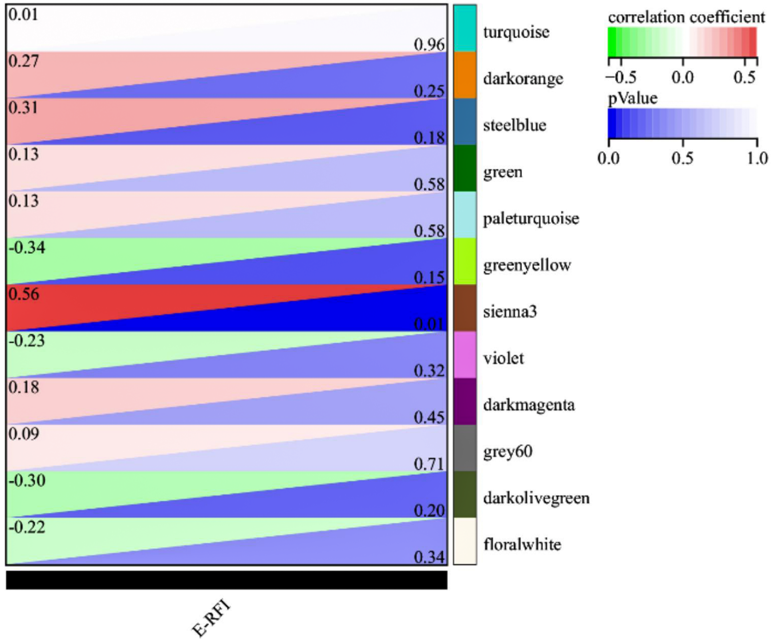
0.01
0.96
0.27
0.25
0.31
0.18
0.13
0.58
0.13
0.58
-0.34
0.15
0.56
0.01
-0.23
0.32
0.18
0.45
0.09
0.71
-0.30
0.20
-0.22
0.34
turquoise
darkorange
steelblue
green
paleturquoise
greenyellow
sienna3
violet
darkmagenta
grey60
darkolivegreen
floralwhite
correlation coefficient
−0.5 0.0 0.5
pValue
0.0 0.5 1.0
E-RFI

Ileum

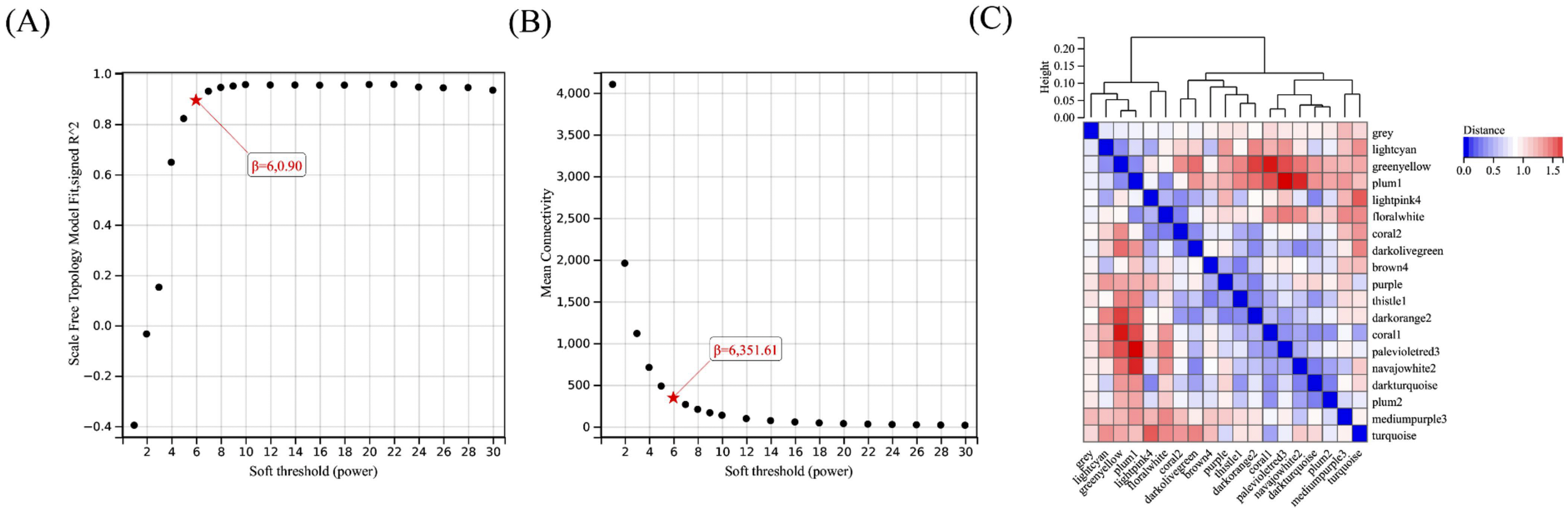

(A)
Scale Free Topology Model Fit,signed R^2
Soft threshold (power)
β=6,0.90
(B)
Mean Connectivity
Soft threshold (power)
β=6,351.61
(C)
Height
Distance
grey
lightcyan
greenyellow
plum1
lightpink4
floralwhite
coral2
darkolivegreen
brown4
purple
thistle1
darkorange2
coral1
palevioletred3
navajowhite2
darkturquoise
plum2
mediumpurple3
turquoise

(D)

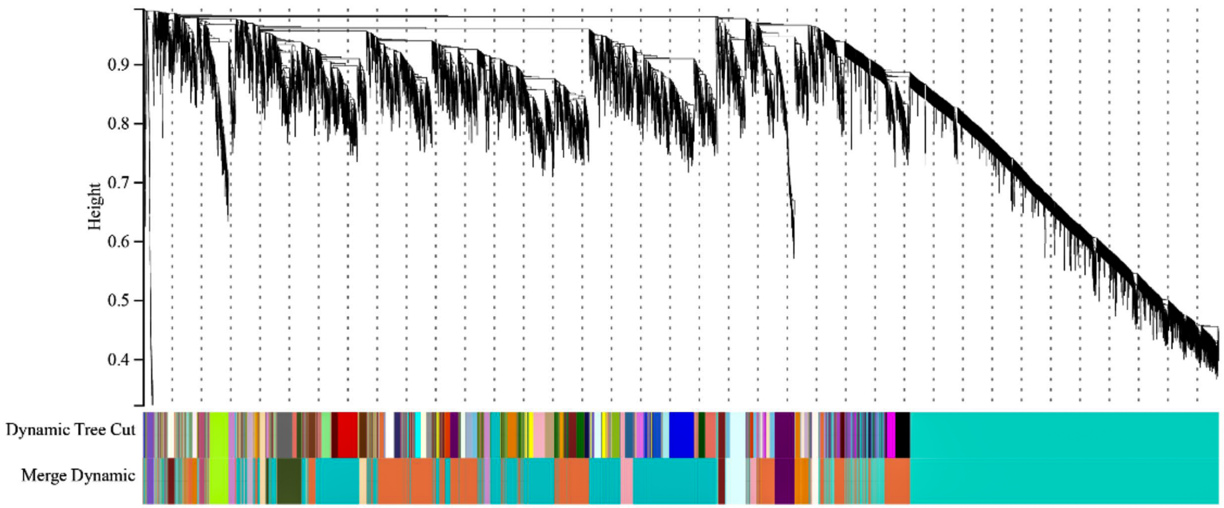
Height
0.9
0.8
0.7
0.6
0.5
0.4
Dynamic Tree Cut
Merge Dynamic

(E)

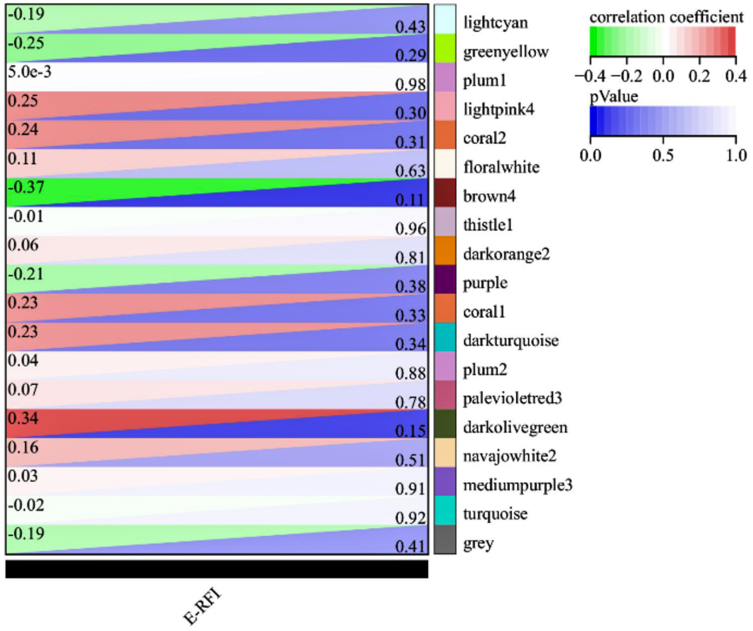
-0.19 0.43
-0.25 0.29
5.0e-3 0.98
0.25 0.30
0.24 0.31
0.11 0.63
-0.37 0.11
-0.01 0.96
0.06 0.81
-0.21 0.38
0.23 0.33
0.23 0.34
0.04 0.88
0.07 0.78
0.34 0.15
0.16 0.51
0.03 0.91
-0.02 0.92
-0.19 0.41
lightcyan
greenyellow
plum1
lightpink4
coral2
floralwhite
brown4
thistle1
darkorange2
purple
coral1
darkturquoise
plum2
palevioletred3
darkolivegreen
navajowhite2
mediumpurple3
turquoise
grey
correlation coefficient
−0.4 −0.2 0.0 0.2 0.4
pValue
0.0 0.5 1.0
E-RFI

Note: (A) Scale independence diagram of each tissue, the horizontal axis represents the soft threshold, the vertical axis represents the scale-free topological model fitting, and β represents the value of the soft threshold; (B) Average connectivity diagram of each tissue, the horizontal axis represents the soft threshold, the vertical axis represents the average connectivity, and β represents the value of the soft threshold; (C) Cluster diagram of feature vectors of each tissue module, the horizontal and vertical axes both represent modules named with different colors; (D) Gene cluster diagram of each tissue; (E) Heat map of the correlation between each tissue module and phenotype, the heat map color represents the positive/negative Pearson correlation coefficient.
